# Xanthones from *Gentianella acuta* (Michx.) Hulten Ameliorate Colorectal Carcinoma via the PI3K/Akt/mTOR Signaling Pathway

**DOI:** 10.3390/ijms24032279

**Published:** 2023-01-23

**Authors:** Meng-Qi Lu, Jing-Ya Ruan, Hui-Min Li, Ding-Shan Yang, Yan-Xia Liu, Mi-Mi Hao, Hai-Yang Yu, Yi Zhang, Tao Wang

**Affiliations:** 1 State Key Laboratory of Component-Based Chinese Medicine, Tianjin University of Traditional Chinese Medicine, 10 Poyanghu Road, West Area, Tuanbo New Town, Jinghai District, Tianjin 301617, China; 2 Tianjin Key Laboratory of TCM Chemistry and Analysis, Tianjin University of Traditional Chinese Medicine, 10 Poyanghu Road, West Area, Tuanbo New Town, Jinghai District, Tianjin 301617, China

**Keywords:** *Gentianella acuta*, xanthone, colorectal carcinoma, bioinformatic analyses, HT29 cells, LoVo cells, PI3K/Akt/mTOR signaling pathway

## Abstract

Colorectal carcinoma (CRC) is a kind of malignant tumor closely related to ulcerative colitis. Xanthone derivatives are one of the most promising therapeutic drugs which have been used in phase I/II clinical trials for cancer therapy. Our previous study indicated that the aerial parts of *Gentianella acuta* Michx. Hulten (GA) was rich in xanthones and showed a good therapeutic effect on ulcerative colitis in mice, suggesting that GA xanthones might have some therapeutic or ameliorative effects on CRC. However, no relevant study has been reported. This study aims to find the effective substances of GA inhibiting CRC and clarify their mechanism. Solvent extraction, column chromatographic separation, and LC-MS analysis were used to characterize the 70% EtOH extract of GA and track xanthones abundant fraction XF. MTT assay was carried out to clarify the activity of GA fractions; the result showed XF to be the main active fraction. LC-MS analysis was executed to characterize XF, 38 xanthones were identified. Network pharmacology prediction, in vitro activity screening, and molecular docking assay were combined to predict the potential mechanism; the PI3K/Akt/mTOR signaling pathway was found to be most important. Western blot assay on the main active xanthones 1,3,5-trihydroxyxanthone (**16**), 1,3,5,8-tetrahydroxyxanthone (**17**), 1,5,8-trihydroxy-3-methoxyxanthone (**18**), and 1,7-dihydroxy-3,8-dimethoxyxanthone (**19**) was used to verify the above prediction; these xanthones were found to inhibit the PI3K/Akt/mTOR signaling pathway, and **17** played a significant role among them through Western blot assay using PI3K/AKT/mTOR agonist IGF-1. In conclusion, this study demonstrated that GA xanthones were effective compounds of GA inhibiting CRC by regulating PI3K/Akt/mTOR signaling pathway transduction, at least. Importantly, 1,3,5,8-tetrahydroxyxanthone (**17**), the most abundant active xanthone in GA, might be a candidate drug for CRC.

## 1. Introduction

Colorectal carcinoma (CRC) is one of the malignant tumors worldwide with the highest morbidity and mortality, and the incidence of it continues to show a younger trend, which is a serious threat to human health [1,2]. At present, laparoscopic colectomy combined with chemoradiotherapy is the primary intervention for CRC, but it can cause adverse effects, such as bowel, bladder, and sexual dysfunction [3]. 5-Fluorouracil (5-FU) is one of the first-line drugs with the longest history in the clinical treatment of CRC. However, its efficacy is diminished by the development of resistance to it in cancer cells, and the response rate for patients with advanced CRC is only approximately 10% [4,5]. In recent years, targeted therapeutic drugs for CRC have emerged. Bevacizumab, a first-line vascular endothelial growth factor, can overcome resistance to 5-FU and other drugs for CRC patients. However, the clinical survival of CRC patients was reduced due to a series of adverse drug reactions, such as gastrointestinal disturbances, hypertension, nephrotoxicity, and impaired wound healing caused by bevacizumab [6,7]. Therefore, there is an urgent need to develop highly effective drugs with low side effects.

Natural products, including Traditional Chinese medicine (TCM) and ethnic medicine, have usually been reported to have mild toxicity and side effects. They are more suitable for the treatment of chronic diseases. In recent years, it has shown good curative effects and unique advantages in cancer treatment, playing an especially important role in preventive health care, reversing multidrug resistance, balancing immunity, weakening the side effects of radiotherapy, and enhancing the efficacy of treatment [8,9,10].

Xanthones are a class of plant phenolic compounds with C6-C1-C6 carbon structure, which are widely found in Clusiaceae, Gentianaceae, Polygalaceae families, and so on [11,12,13]. They have been reported to display diverse pharmacological effects, such as anti-tumor, anti-inflammatory, anti-bacterial, anti-viral, hepatoprotection, and hypoglycemia [14,15,16,17]. The most studied xanthone, α-mangostin, can interfere with the development of colon, breast, lung and other cancers, including induction of growth arrest and apoptosis, and inhibition of cancer cell angiogenesis and metastasis [18]. Meanwhile, several xanthone derivatives are currently being used in phase I/II clinical trials for cancer therapy, and they can induce cancer cell death by acting on topoisomerase II, inducing autophagy, inhibiting cell proliferation, promoting apoptosis, and blocking cell cycle [19,20,21].

*Gentianella acuta* Michx. Hulten (GA) is an annual herb of *Gentianella* genus, Gentiananceae family. It is used for treating jaundice, headache, fever, cholecystitis, angina pectoris, and traumatic infections in Mongolian medicine and had pharmacological effects of anti-arrhythmia, anti-depression, anti-inflammatory, anti-tumor, and improvement of insulin resistance [22]. Our previous study suggested that the main constituents in GA were xanthones and iridoids [23]. In addition, GA can be considered as a candidate drug for the treatment of ulcerative colitis (UC) since the 70% EtOH extract of GA as well as its main compound, desmethylbellidifolin (1,3,5,8-tetrahydroxyxanthone) could attenuate the TNBS-induced inflammatory response in vivo and dilate the colonic muscle [24]. 1,3,5,8-tetrahydroxyxanthone can maintain the intestinal barrier function, block the production of inflammation-promoting factors, and balance the intestinal flora in mice, thus effectively improving the symptoms of UC [25]. A worldwide study of new CRC cases each year has found that patients with inflammatory bowel disease (IBD) such as Crohn’s disease (CD) and UC, are at high risk of developing CRC. At the same time, the risk of CRC increases with the duration of IBD and the degree of inflammation [26,27]. Abovementioned findings suggest that GA may have some therapeutic or ameliorative effects on CRC. However, no data about it have been reported.

First, to explore the effective components of GA that interfere with CRC and their mechanism, 70% EtOH extract of GA was treated with solvent extraction and column chromatography (CC) separation to obtain different fractions, and LC-MS analysis was used to identify their chemical composition. Second, in vitro anti-CRC bioassay was performed to definite the active fractions. Third, a network pharmacology analysis was introduced to predict the potential targets and related signaling pathways of the active fraction intervention in CRC. Fourth, the constituents in them were screened for in vitro anticancer activity. Finally, the anti-CRC mechanisms of the active compounds were verified by molecular docking and Western blot assay.

## 2. Results

### 2.1. Isolation and Chemical Composition Analysis of GA Extracts

**Figure 1:**
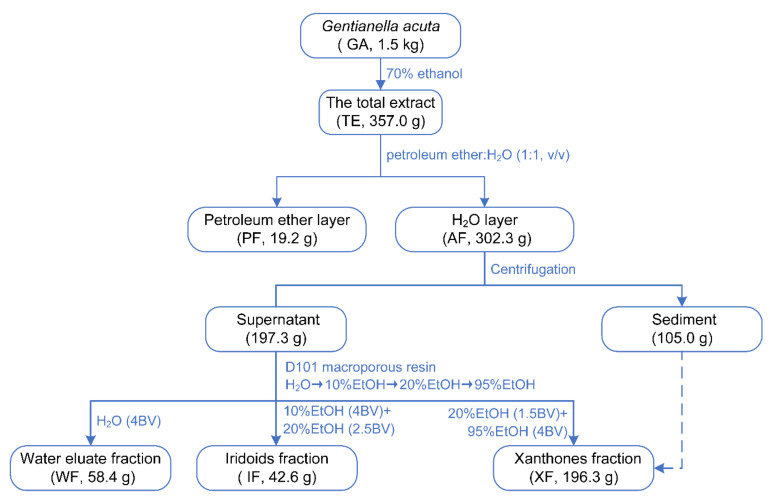
Extraction and separation process of *G. acuta* component groups.

The dried GA was extracted under reflux with 70% EtOH. A residue (TE) was provided after removal of the solvent under reduced pressure. TE was partitioned with H_2_O-PE (1:1, *v*/*v*) to obtain the PE layer (PF) and the H_2_O layer (AF). AF was centrifugated to obtain supernatant and sediment. The supernatant was successively subjected to D101 macroporous resin CC and gradient eluted with H_2_O, 10% EtOH, 20% EtOH, and 95% EtOH. According to the LC-MS analysis result, the water fraction (WF), iridoids fraction (IF), and xanthones fraction (XF) were obtained (Figure 1).

**Figure 2:**
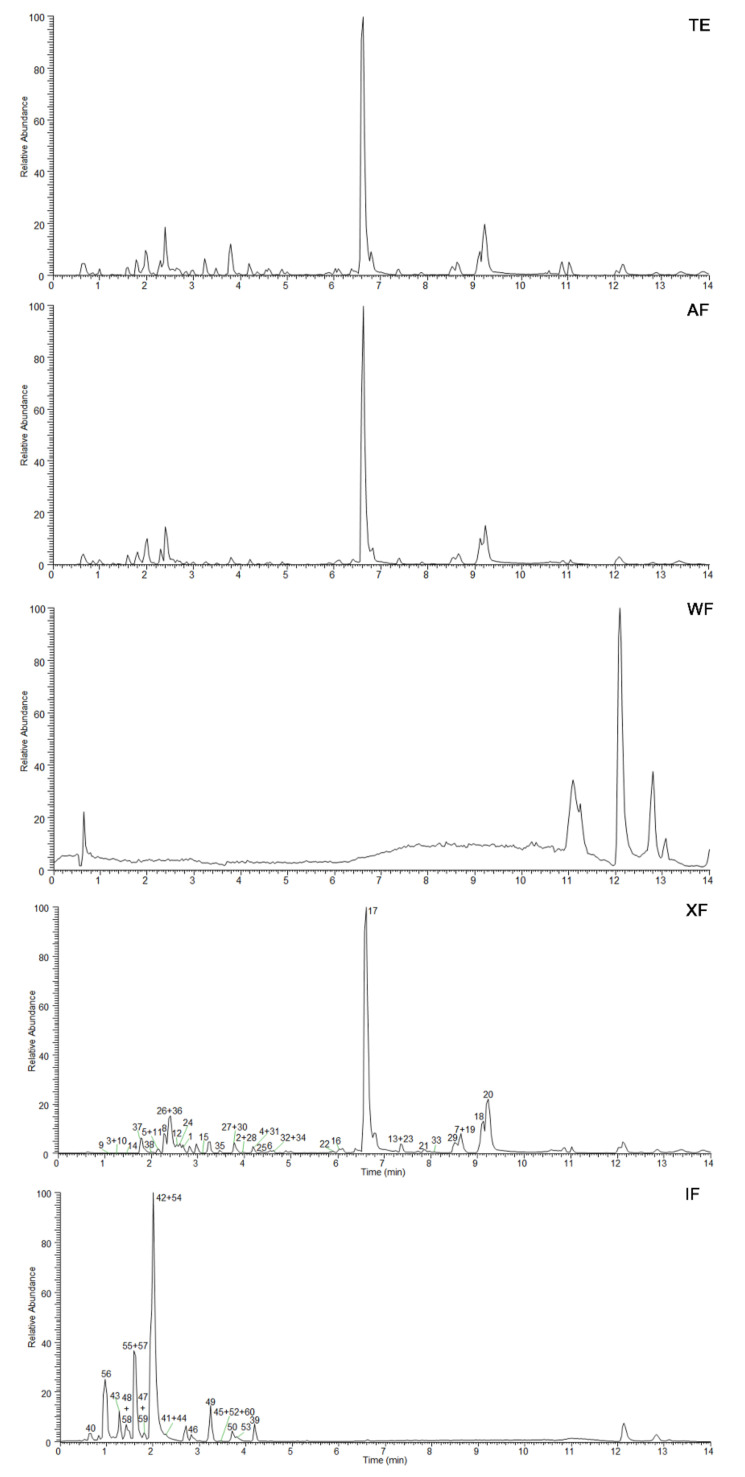
BPC chromatograms of TE, AF, WF, IF and XF, respectively. Chromatographic conditions: Column: ACQUITY UPLC^®^ BEH C18 (1.8 µm, 2.1 × 100 mm); mobile phase: FA-H_2_O (1:1000, *v*/*v*) (A) and ACN (B) in the gradient program: 0–6 min, 15–45% B; 6–9 min, 45% B, 9–10 min, 95% B; 10–14 min, 95% B; column temperature: 30 °C; flow rate: 0.4 mL/min; injection volume: 2 µL. Mass spectrometry conditions: Ion source: heat electrospray ion source (HESI source); capillary voltage: 3.2 kV; capillary temperature: 320 °C; ion source temperature: 300 °C; sheath gas (N_2_): 40 L/h; auxiliary gas (N_2_): 10 L/h; normalized collision energy (NCE): 35 V; scan mode: full scan; scan range: 100–1500 *m*/*z*; detection time: 14 min; ESI acquisition mode: negative ion mode.

TE, AF, WF, IF, and XF were qualitatively analyzed by UHPLC-Q Exactive-Orbitrap MS technology combined with retention time, as well as primary and secondary mass spectrometry information. In the results, little difference was displayed between TE and AF (Figure 2), suggesting that the small molecules in GA were mainly concentrated in AF. After subsequent separation, WF, XF, and IF were obtained from AF. According to the analysis results in Figure 2 and the literature report [23], XF and IF were identified as the enrichment fraction of major components in GA. By comparing the retention time and mass spectrometry information of chemical constituents in them with those of references (Table S1, Figure S1), 38 xanthones and 20 iridoids were clearly identified from XF and IF, respectively. Compound **51** was not detected because of its low content. These results indicated that the extraction and isolation process used in the experiment was well-designed and could adequately separate the iridoids and xanthones in GA. In addition, PF was not characterized qualitatively since it did not contain xanthones and iridoids (analyzed by thin-layer chromatography) and had a low proportion compared with other fractions. It was worth mentioning that 1,3,5,8-tetrahydroxyxanthone (**17**) was the main constituent of XF.

### 2.2. Intervention Effect of Different Fractions on CRC Cells

HT29 (MSS type, KRAS wild type, BRAF mutation, TP53 mutation) and LoVo (MSI type, KRAS mutation, BRAF wild type, TP53 wild type) [28,29] cells with large genetic differences were selected as in vitro screening models. 3-(4,5-Dimethyl-2-thiazolyl)-2,5-diphenyl tetrazolium bromide (MTT) assay was used to evaluate the inhibitory effects of TE, PF, AF, WF, IF, and XF for exploring the effective fraction in GA intervention on CRC. The results suggested that TE, PF, AF, and XF showed a concentration-dependent inhibitory effect. Among them, XF displayed the strongest activity, whose IC_50_ values on HT29 and LoVo cells were 85.6 ± 2.49 and 76.3 ± 2.16 μg/mL, respectively (Table 1). It was tentatively concluded that the active compounds of GA intervention on CRC were concentrated in XF. Therefore, the two cell lines were used to further explore the effect of XF in the following experiments.

### 2.3. XF Inhibits the Proliferation of CRC Cells

**Figure 3:**
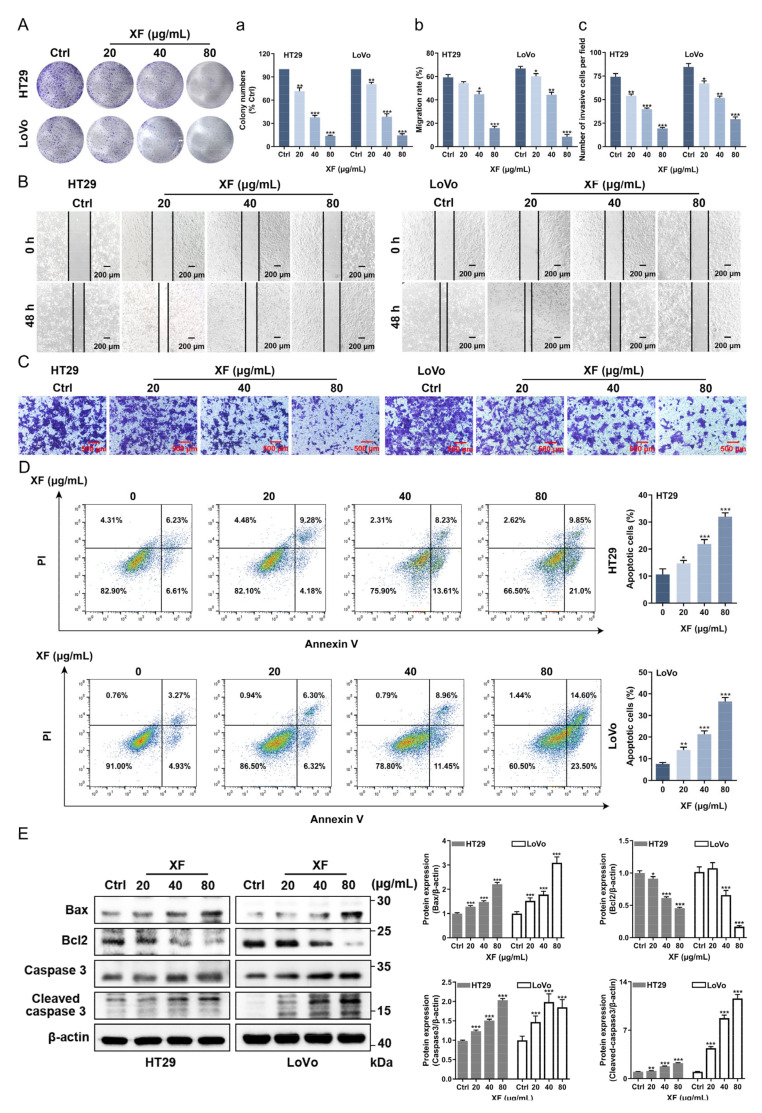
Effect of XF on HT29 and LoVo cells. (**A**,**a**) Effect of XF on HT29 and LoVo cells proliferation; (**B**,**b**) Effect of XF on HT29 and LoVo cells migration; (**C**,**c**) Effect of XF on HT29 and LoVo cells invasion; (**D**) Representative results of Annexin V-FITC/PI staining of HT29 and LoVo cells treated with XF for 24 h; (**E**) Apoptosis related protein expressions of Bax, Bcl2, caspase 3, and cleaved-caspase 3 in XF-treated CRC cells. Data are presented as the means ± SEM, *n* = 3. * *p* < 0.05, ** *p* < 0.01, *** *p* < 0.001.

Clone formation assay can reflect the dependence and sensitivity of cells to drug concentration and can then be used to elucidate the inhibitory effect of drugs on cell proliferation. Compared with the control group, the proliferation process of HT29 and LoVo cells was significantly inhibited with the increase of XF administration concentration at 20, 40, and 80 μg/mL (Figure 3A,a).

### 2.4. XF Inhibits the Migration and Invasion of CRC Cell

The cell scratch assay was performed to study the inhibitory effect of XF on HT29 and LoVo cells migration. The results showed that the scratch healing ability of HT29 and LoVo cells were significantly reduced 48 h after XF administration compared with the control group (Figure 3B,b), suggesting that XF could inhibit the migration of CRC cells. Meanwhile, the effect of XF on HT29 and LoVo cells invasion was detected by Transwell assay. It was found that 80 μg/mL XF could significantly inhibit the invasion behavior of two CRC cell lines from the upper chamber to the lower chamber through Matrigel 48 h after being treated with XF (Figure 3C,c), which indicated that XF could inhibit CRC cell invasion. Moreover, the results showed that XF inhibited the migration and invasion of CRC cells in a concentration-dependent manner at the concentrations of 20, 40, and 80 μg/mL.

### 2.5. XF Promotes the Apoptosis of CRC Cells

The induction of apoptosis by XF on CRC cell lines HT29 and LoVo cells was assessed with Annexin V-FITC/PI assay. As shown in Figure 3D, the proportion of Annexin V-positive cells increased with the increase of XF administration concentration, especially 80 μg/mL of XF, significantly increasing the percentages of apoptotic cells to 31.98% ± 3.36% and 36.50% ± 3.47% in two kinds of cells, respectively.

Proteins involved in the process of apoptosis mainly include caspase, B-cell lymphoma-2 (Bcl-2) family proteins, such as Bcl-2, Bcl-2-associated X protein (Bax), and inhibitors of apoptosis proteins (IAPs), etc. Among them, caspase belongs to cysteine proteases, and its active site contains cysteine residues, which can specifically cleave peptide bonds on aspartic acid residues, thus causing cell apoptosis [30]. The Bcl-2 protein family can activate caspases through oligomerization and trigger downstream enzyme-linked reactions, leading to cell apoptosis [31]. As a pro-apoptotic member of the Bcl-2 family, Bax plays the role of the molecular switch through the mitochondrial signaling pathway. Its high expression can antagonize the protective effect of Bcl-2 on cells and make cells tend to die [32]. As shown in Figure 3E, the Western blot assay suggested XF could significantly up-regulate the expression of apoptosis-related proteins, caspase-3, cleaved-caspase-3, and Bax and down-regulate the expression of Bcl-2 in both CRC cells lines. Moreover, the effects were displayed in a concentration-dependent manner at 20, 40, and 80 μg/mL.

### 2.6. Xanthones in XF Inhibited Tumor Cell Proliferation In Vitro

The abovementioned experiments suggested that XF was the main pharmacodynamic component of GA in the intervention of CRC. Based on the results, we further evaluated the in vitro activity of the 38 xanthones (**1**–**38**) identified from XF against HT29 and LoVo cells proliferation by MTT assay. It was found that compounds 1,3,5-trihydroxyxanthone (**16**), 1,3,5,8-tetrahydroxyxanthone (**17**), 1,5,8-trihydroxy-3-methoxyxanthone (**18**), 1,7-dihydroxy-3,8-dimethoxyxanthone (**19**), 1,7-dihydroxy-3,4-dimethoxyxanthone (**22**), and 1,7-dihydroxy-3,4,8-trimethoxyxanthone (**23**) showed significant inhibitory effect on the proliferation of both CRC cells lines, and their IC_50_ was less than 100 μM (Table 2), which tentatively indicated that they had the potential to be further developed as anti-CRC drugs.

**Figure 4:**
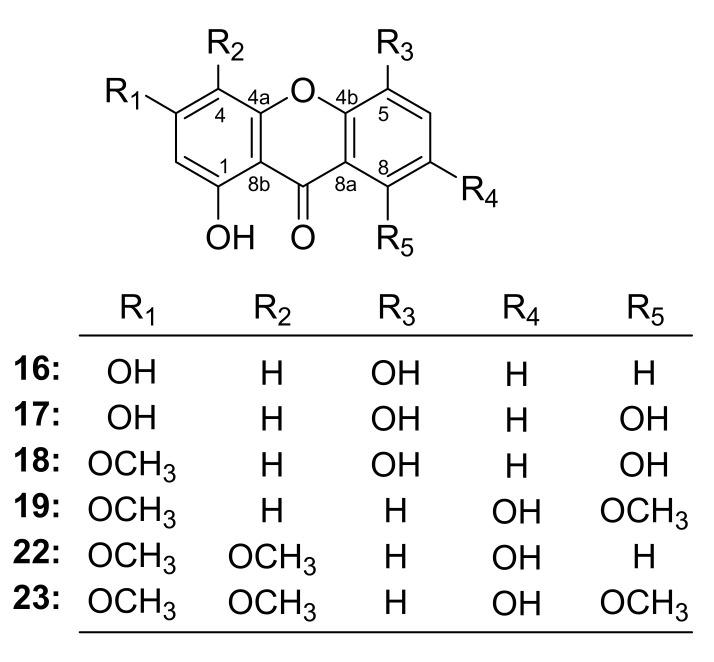
The structures of xanthones **16**–**19**, **22** and **23**.

By comparing the structures of the above active xanthones (Figure 4), the structure-activity relationships (SARs) could be concluded that:

(1) The oxygenated substitution at C-4 in the xanthone might be the key group for its antiproliferative activity in tumor cells (**22**, **23** vs. **16**–**19**).
(2) The activity of xanthone substituted in C-3 by hydroxyl was superior to that substituted by methoxy (**17** vs. **18**).

The SARs would provide a reference for the structural modification of xanthones in the development of anti-CRC drugs.

### 2.7. Active Xanthones May Prevent CRC through PI3K/Akt/mTOR Pathway by Network Pharmacology Prediction

#### 2.7.1. Components-Disease Genes Associated with CRC

**Figure 5:**
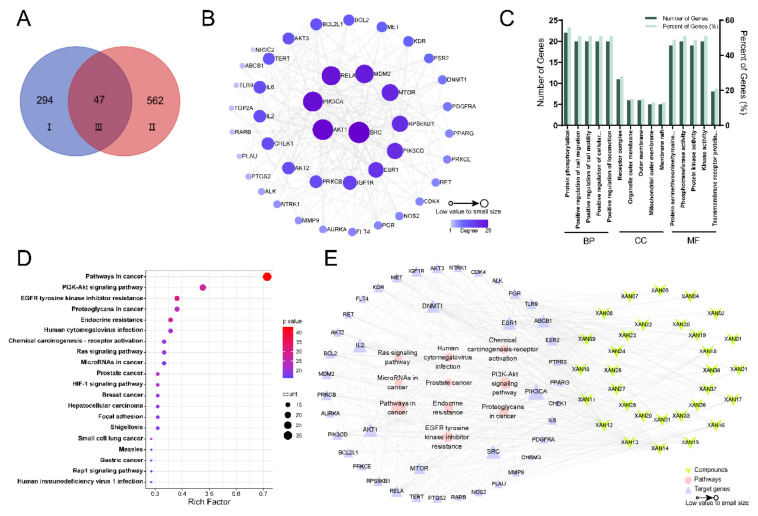
The network pharmacology analysis for the anti-CRC activities of xanthones. (**A**) Venn diagram of the targets of xanthones-CRC (I: targets related to xanthones; II: targets related CRC; III: the intersecting targets for xanthones and CRC); (**B**) PPI network of the intersecting targets for xanthones and CRC. (**C**) Annotation analysis of Gene Ontology; (**D**) The visual results of KEGG signal pathway; (**E**) Component-target-pathway interaction network diagram.

Network pharmacological analysis combined with pharmacological or molecular biological experimental validation is an effective method to study the complex mechanism of in vivo drug action. To further elucidate the mechanism of the six active xanthones, network pharmacology was first used to predict their potential targets. A literature review showed that they have been poorly studied in CRC; to fully predict their potential targets, the target collection in this study was set to be the 38 xanthones obtained from GA [23]. Detailed information on compound names, structural formulae and SMILES strings could be found in Table S2. The target information of xanthones and CRCs was accurately retrieved as described in Section 4.9.1 and Section 4.9.2. In total, 294 and 562 gene targets were predicted to be most relevant to GA xanthones and CRC, respectively. It is worth noting that when encountering new compounds that are not yet included in the database, the structural similarity score value can be adjusted to be greater than 0.9; the closer it is to 1, the more similar it is to the structure of the target compound; thus, it can help to predict the potential biological activity of some new compounds. Finally, the compound targets were mapped to the disease targets by a Venn diagram to obtain 47 overlapping targets (Figure 5A, Table S3).

#### 2.7.2. Construction of PPI Network Diagram

The 47 overlapping targets obtained in Section 2.7.1 were imported into the STRING database to construct a PPI network interacting GA xanthones and CRC-related targets by Cytoscape 2.7.1 software, and the related topological parameters were analyzed. The network graph had 42 nodes and 225 edges, with 5 discrete nodes removed. The degree value was a key parameter to measure the importance of nodes. The greater connectivity was sustained by the higher degree value and the larger node shape, which indicated that the node was more likely to be a potential target (Figure 5B). The top 10 core targets were non-receptor tyrosine kinase (SRC) (degree = 25), Akt1 (degree = 24), PIK3CA (degree = 23), RELA (degree = 22), MDM2 (degree = 21), mTOR (degree = 19), RPS6KB1 (degree = 18), PIK3CD (degree = 18), ESR1 (degree = 17), and IGF1R (degree = 15) in turn (Table S4). Among them, CRC protein can be activated by multiple signal transduction pathways and is associated with the occurrence and development of a variety of tumors [33], which is with weak specificity for CRC. Akt1, PIK3CA, and mTOR are closely associated with the development of CRC [34].

#### 2.7.3. GO Functional Annotation and KEGG Pathway Enrichment Analysis

Compared to the DAVID database, Metascape updates the database monthly to better meet our requirements for the accuracy of results [35]. Therefore, the predicted targets were imported into Metascape software for GO biological function analysis to elucidate the mechanism of xanthones against CRC, and the results were shown in Figure 5C, which suggested that the target genes might be involved in biological processes, such as protein phosphorylation and cell migration, whose functional location might be located in the receptor complexes, organelle outer membranes, and extracellular membranes. Meanwhile, the molecular functions might include the protein kinase activity of serine/threonine/tyrosine.

A total of 141 pathways were enriched by KEGG pathway analysis. According to the rich factor ranking, 20 major related pathways were screened out, and the bubble diagram was used to visualize the enrichment results of the KEGG pathway. The larger the bubble shape, the more related targets enriched in this pathway, the redder the bubble color, and the smaller the *p*-value (Figure 5D). The results indicated that the pathways in the cancer signaling pathway (with 30 associated genes), PI3K/Akt signaling pathway (with 20 associated genes) and EGFR tyrosinase inhibition resistance (with 16 associated genes) were the main signaling pathways. It was found that PI3K/Akt signaling pathway was closely related to the proliferation and apoptosis of CRC according to the literature review [34], suggesting the xanthones in XF might interfere with this pathway to affect the development of CRC.

#### 2.7.4. Analysis of the Results of Components-Targets-Pathways Network Construction

The component-target-pathway association table was imported into Cytoscape 3.7.1 software to construct the “component-target-pathway” association network of GA. As shown in Figure 5E, the central triangle was the target gene; a larger suggested node indicated a higher connectivity. The top seven target genes were PIK3CA, Akt1, SRC, IL2, DNMT1, ESR1, and mTOR. PI3K3CA is involved in the regulation of cellular growth, transformation, adhesion, apoptosis, survival, and motility. In some situations, the PI3K/Akt pathway gets altered due to a mutation in the PIK3CA gene producing the oncogenic event in human malignancy [36]. In addition, all three Akt subtypes (Akt1, Akt2, Akt3) were present in our analysis. Among them, Akt1 showed the highest association with CRC. AKT1 is mainly regulated by the upstream gene PI3K and is also a key regulator of the PI3K/Akt signaling pathway [37]. Meanwhile, the phosphorylation level of mTOR is a tumor marker of CRC and an important regulatory protein in the PI3K/Akt/mTOR signaling pathway, which mainly regulates cell survival, proliferation and apoptosis, and other physiological functions [38]. Large numbers of reports have found that PI3K/Akt/mTOR pathway is abnormally activated in the cancer environment and plays a key regulatory role in the proliferation and apoptosis of cancer cells [39]. In conclusion, we hypothesized that GA xanthones might intervene in CRC by regulating the PI3K/Akt/mTOR pathway.

### 2.8. Analytical Results of Molecular Docking

**Figure 6:**
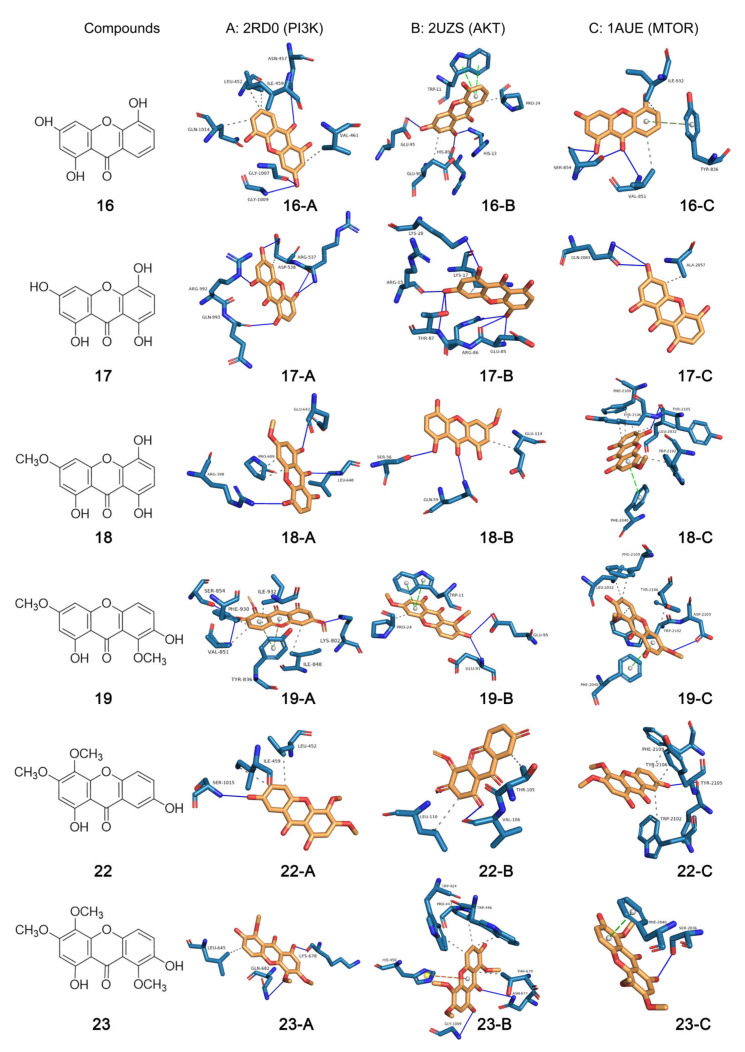
Molecular docking diagram of proteins. (**A**) 2RD0-PIK3CA, (**B**) 2UZS-Akt1, (**C**) 1AUE-mTOR and ligands (xanthones **16**–**19**, **22** and **23**).

Molecular docking is widely used in the study of protein-ligand interactions, usually starting from the three-dimensional structures of known ligands and receptors, and then using computer-fitted site docking to predict the binding conformation and binding free energy of small molecules to target proteins [40]. To confirm the speculation in Section 2.7, we adopted the molecular docking method to perform molecular docking with the key proteins of PI3K/Akt/mTOR pathway [PI3K (PIK3CA, PDB ID: 2RD0, 3.05 Å), Akt (Akt1, PDB ID: 2UZS, 2.46 Å), mTOR (PDB ID: 1AUE, 2.06 Å)] for the six active xanthones [1,3,5-trihydroxyxanthone (**16**), 1,3,5,8-tetrahydroxyxanthone (**17**), 1,5,8-trihydroxy-3-methoxyxanthone (**18**), 1,7-dihydroxy-3,8-dimethoxyxanthone (**19**), 1,7-dihydroxy-3,4-dimethoxyxanthone (**22**), and 1,7-dihydroxy-3,4,8-trimethoxyxanthone (**23**)]. The binding conformation of molecular docking and the parameter information are shown in Figure 6 and Table 3, respectively. All affinity values were less than −5 kcal/mol (Table 3), indicating that the receptor and ligand could produce good affinity for each other. These results suggested that the active xanthones affected intermolecular signaling through their interactions with PIK3CA, Akt1, and mTOR, thereby playing a role in interfering with CRC cells.

### 2.9. Results of Western Blotting Assay

**Figure 7:**
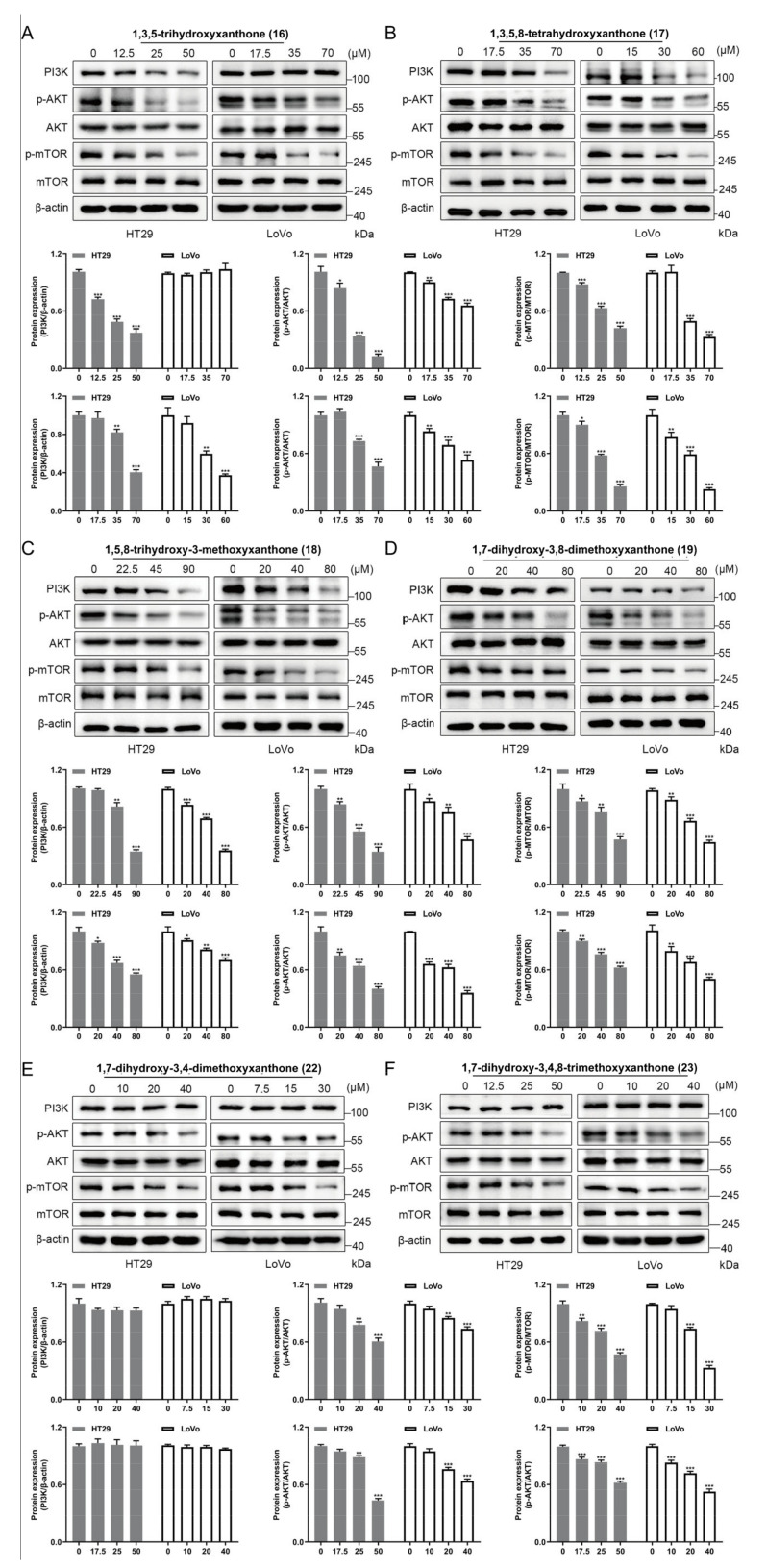
The regulation of xanthones **16**–**19**, **22** and **23** on PI3K/Akt/mTOR signaling pathway in vitro. Expression and statistics of PI3K, p−Akt (Ser473)/Akt, p−mTOR (Ser2448)/mTOR proteins detected after treatment of HT29 and LoVo cells with compounds (**A**) 1,3,5−trihydroxyxanthone (**16**); (**B**) 1,3,5,8−tetrahydroxyxanthone (**17**); (**C**)1,5,8−trihydroxy-3-methoxyxanthone (**18**); (**D**) 1,7−dihydroxy−3,8−dimethoxyxanthone (**19**); (**E**) 1,7−dihydroxy−3,4−dimethoxyxanthone (**22**); (**F**) 1,7−dihydroxy−3,4,8−trimethoxyxanthone (**23**), respectively. Values represent the mean ± SEM of three determinations. * p < 0.05; ** p < 0.01; *** p < 0.001 (Differences between compound-treated group and control group). *n* = 3.

Though the molecular docking method has been widely used in the prediction of ligand-protein interaction, it can only predict the strength of ligand-protein binding ability not determine whether the ligands activate or inhibit the receptors. Therefore, Western blotting assay was performed to investigate the effects of xanthones **16**–**19**, **22**, and **23** on the PI3K/Akt/mTOR pathway by using HT29 and LoVo cells. The experimental results showed that 1,3,5-trihydroxyxanthone (**16**) could dose-dependently down-regulate the expression levels of PI3K, p-Akt, and p-mTOR in HT29 cells, and it showed no significant effect on the expression of PI3K in LoVo cells (Figure 7A). Meanwhile, 1,3,5,8-tetrahydroxyxanthone (**17**), 1,5,8-trihydroxy-3-methoxyxanthone (**18**), and 1,7-dihydroxy-3,8-dimethoxyxanthone (**19**) could reduce the expression of PI3K, p-Akt, and p-mTOR in a concentration-dependent manner in two CRC cell lines (Figure 7B–D). On the other hand, 1,7-dihydroxy-3,4-dimethoxyxanthone (**22**) and 1,7-dihydroxy-3,4,8-trimethoxyxanthone (**23**) did not significantly regulate PI3K protein but significantly down-regulated the expression levels of Akt and mTOR proteins in two cells (Figure 7E,F). These results basically verified those predicted by network pharmacology and were generally consistent with those simulated by molecular docking.

**Figure 8:**
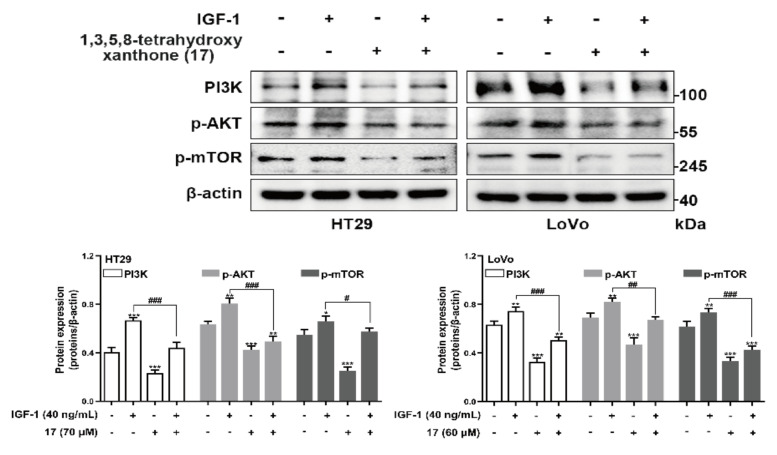
Western blot verification of 1,3,5,8−tetrahydroxyanthrone (**17**) intervening CRC by PI3K/Akt/mTOR signaling pathway. CRC cells were treated with IGF−1 (40 ng/mL) for 24 h, 1,3,5,8−tetrahydroxyxanthone (**17**) was treated with 70 μM for HT29 cells and 60 μM for LoVo cells. Expression and statistics of PI3K, p−Akt (Ser473), p−mTOR (Ser2448) proteins detected after treatment of HT29 and LoVo cells with compound **17**. Data are presented as the means ± SEM, * *p* < 0.05, ** *p* < 0.01, *** *p* < 0.001 (Differences between compound-treated group, IGF−1-treated group, as well as (compound + IGF−1)-treated group and control group). ^#^ *p* < 0.05, ^##^ *p* < 0.01, ^###^ *p* < 0.001 (Differences between (compound + IGF−1)-treated group and IGF−1-treated group). *n* = 3.

To prove the anti-CRC mechanism of the compounds was related to the PI3K/Akt/mTOR pathway, agonist IGF-1 of PI3K/AKT/mTOR was used for a Western blot assay to test the related effects of the main ingredient 1,3,5,8-tetrahydroxyanthrone (**17**) in XF and GA (Figure 2). In the results, IGF-1 increased the protein expressions of PI3K, p-AKT, and p-mTOR compared with the control group, while compound **17** could significantly inhibit the increasement (Figure 8).

## 3. Discussion

In the treatment of cancer, especially CRC, the toxic side effects of chemotherapeutic drugs as well as drug resistance limit efficacy [3,6]. Finding anti-cancer drugs from natural products is one of the ways to solve this problem. Some natural xanthones have been reported to have good anti-CRC effects. For example, α-mangostin had chemopreventive effects in short-term CRC animal models and might inhibit the development and progression of CRC [41]. Another xanthone, gambogic acid was clarified to interfere with the migration and invasion of CRC by mediating microRNA-21-activated phosphatase [42]. What’s more, it has shown a better safety profile in clinical phase II studies of advanced malignancies at doses of 45 mg/m^2^ and a higher rate of disease control in patients tested at 1–5 d [43]. The above research provides a direction for us to find anti-CRC drugs from natural products. Our current research firstly found xanthones enriched fraction XF of GA could affect the occurrence and development of CRC by inhibiting the proliferation, migration, and invasion, as well as promoting the apoptosis of HT29 and LoVo cells.

Natural medicines contain complex and diverse components, which can be used to treat diseases more comprehensively by regulating multiple signaling pathways. However, it is difficult to conduct comprehensive and systematic research from the overall to the molecular level because of the unclear material basis and potential mechanisms, and the lack of safety evaluation systems. Fortunately, the research method of network pharmacology has been continuously applied in the process of exploring the mechanism of the action of herbal and botanical medicines. This study helped us to rapidly predict that the xanthones might intervene with CRC through PI3K/Akt/mTOR signaling pathway.

PI3K/Akt/mTOR pathway are activated through several cellular stimuli and control essential functions of cells. It is involved in numerous biological processes, such as the cell cycle, apoptosis, and autophagy, through the transduction of intra- and extracellular signals [44]. They are frequently seen as a single unique pathway that interacts with numerous other pathways including mitogen-activated protein kinase (MAPK), wingless/integrated (Wnt), and c-Jun N-terminal kinase (JNK), because of their close interconnections [45]. Understanding how to control cell proliferation and the apoptotic pathways that are connected to cell death is essential for understanding how cancer cells function. Normal human physiology depends on the PI3K/Akt/mTOR signaling system, and changes in its control may result in a variety of malignancies. As a result, the PI3K/Akt pathway is a critical therapeutic target for the development of novel anticancer drugs since it is associated with a variety of human cancers [46]. As one of the key upstream molecules of the PI3K/Akt/mTOR pathway, PI3K can activate the cascade reaction through the phosphorylation of phosphati-dylinositol-4,5-bisphosphate (PIP2) to form phosphati-dylinositol-3,4,5-trisphosphate (PIP3). PIP3 can recruit pyruvate phosphate dikinase (PPDK1) and Akt to the cell membrane, resulting in PDK1-dependent activation of Akt. Akt can phosphorylate and inhibit cell apoptosis, arrest cyclin, and activate mTOR, thereby affecting the expression of downstream genes such as hypoxia-inducible factor-1*α*, and promoting the occurrence and development of tumors [39]. In mammalian cells, mTOR is a serine/threonine kinase that is widely expressed. It is a crucial protein essential for life that has been conserved throughout evolution from yeast form to human form. IGF-1 and its receptor (IGFR-1), vascular endothelial growth factor and related receptor (VEGFR), and human epidermal growth factor and human epidermal growth factor receptor (HER), among others, function as positive growth regulators and send signals to mTOR via PI3K-Akt [47]. Once PI3K/Akt/mTOR is activated, the proliferation and invasion of tumor cells will be out of control. Therefore, blocking the PI3K/Akt/mTOR signaling pathway can effectively inhibit tumorigenesis and progression.

In this study, the xanthones, 1,3,5-trihydroxyxanthone (**16**), 1,3,5,8-tetrahydroxyxanthone (**17**), 1,5,8-trihydroxy-3-methoxyxanthone (**18**), 1,7-dihydroxy-3,8-dimethoxyxanthone (**19**), 1,7-dihydroxy-3,4-dimethoxyxanthone (**22**), and 1,7-dihydroxy-3,4,8-trimethoxyxanthone (**23**) were elucidated to be effective compounds through the combination of methods including in vitro activity screening and molecular docking firstly. As expected, compounds **16**–**19** could down-regulate the expression of PI3K and the phosphorylation of Akt and mTOR in a concentration-dependent manner in Western blot assay. These results proved that xanthones in GA intervened in CRC via PI3K/Akt/mTOR pathway, which was verified by the Western blot assay using PI3K/AKT/mTOR agonist IGF-1. Our research firstly found xanthones from GA could ameliorate the occurrence and development of CRC in vitro. While it cannot be ignored that compared to some clinical medication, the IC_50_ value of them is higher, which indicated that to further develop them in treating CRC, structural modification for improving their solubility is in urgent need.

Meanwhile, anti-oxidant may be another useful strategy for inhibiting CRC; the abundant phenolic hydroxyl groups in the structure of xanthones should be important groups for scavenging free radicals and reactive oxygen species [48]. In light of that, the anti-oxidant ability of GA xanthones should provide more ideas for the treatment of cancer [49].

## 4. Materials and Methods

### 4.1. Materials

The whole plant of *G. acuta* was collected in Mohe County, Daxinganling Region, Heilongjiang Province, China. It was identified by Prof. Lin Ma of Tianjin University of Traditional Chinese Medicine as *Gentianella acuta* (Michx.) Hulten. The voucher specimens (20210418) were deposited at Tianjin University of Traditional Chinese Medicine and the State Key Laboratory of Component-based Chinese Medicine.

38 Xanthone reference compounds, 2-hydroxyethyl-3-carboxymethyl-5,7-dihydroxychromone (**1**), gentichromone A_2_ (**2**), gentichromone A_3_ (**3**), 1,3,5,8-tetrahydroxyxanthone 3-*O*-β-d-glucopyranoside (**4**), 1,3,5,8-tetrahydroxyxanthone 1-*O*-β-d-glucopyranosyl(1→6)-β-d-glucopyranoside (**5**), 1,3,8-trihydroxy-4-methoxyxanthone 5-*O*-β-d-glucopyranoside (**6**), (5*R*,8*S*)-1,3,5,8-tetrahydroxy-5,6,7,8-tetrahydroxanthone (**7**), (5*S*,8*S*)-1,3,5,8-tetrahydroxy-5,6,7,8-tetrahydroxanthone (**8**), (5*R*,8*S*)-1-*O*-β-d-glucopyranosyl-1,3,8-trihydroxy-5,6,7,8-tetrahydroxanthone (**9**), (5*R*,8*S*)-3-*O*-β-d-glucopyranosyl-1,3,8-trihydroxy-5,6,7,8-tetrahydroxanthon (**10**), (5*R*,8*S*)-8-*O*-β-d-glucopyranosyl-1,3,8-trihydroxy-5,6,7,8-tetrahydroxanthone (**11**), (5*R*,8*S*)-8-*O*-β-d-xylopyranosyl-1,3,8-trihydroxy-5,6,7,8-tetrahydroxanthone (**12**), 1-*O*-β-d-glucopyranosyl-3,5,8-trihydroxyxanthone (**13**), norswertianolin (**14**), amarellin B (**15**), 1,3,5-trihydroxyxanthone (**16**), 1,3,5,8-tetrahydroxyxanthone (**17**), 1,5,8-trihydroxy-3-methoxyxanthone (**18**), 1,7-dihydroxy-3,8-dimethoxyxanthone (**19**), 1,3,8-trihydroxy-4,5-dimethoxyxanthone (**20**), 1,3,8-trihydroxy-4,7-dimethoxyxanthone (**21**), 1,7-dihydroxy-3,4-dimethoxyxanthone (**22**), 1,7-dihydroxy-3,4,8-trimethoxyxanthone (**23**), 1-*O*-β-d-glucopyranosyl-3,5,8-trihydroxyxanthone (**24**), 1,3,8-trihydroxyxanthone-5-*O*-β-d-glucopyranoside (**25**), 8-*O*-β-d-glucopyranosyl-1,3,5,8-trihydroxyxanthone (**26**), swertianolin (**27**), triptexanthoside A (**28**), 3,8-dimethoxy-7-hydroxyxanthone 1-*O*-β-d-glucopyranoside (**29**), 1-*O*-[β-d-xylopyranosyl(1→6)-β-d-glucopranosyl]-7-hydroxyl-3,8-dimethoxyxanthone (**30**), 3,7,8-trimethoxyxanthone 1-*O*-β-d-glucopyranoside (**31**), 1-*O*-[β-d-xylopyranosyl(1→6)-β-d-glucopranosyl]-3,7,8-trimethoxyxanthone (**32**), 1-hydroxy-3,4-dimethoxyxanthone 7-*O*-β-d-glucopyranoside (**33**), 3,8-dihydroxy-4,5-dimethoxyxanthone 1-*O*-β-d-glucopyranoside (**34**), 1-*O*-[β-d-glucopyranosyl(1→6)-β-d-glucopyranosyl]-3,8-dihydroxy-4,5-dimethoxyxanthone (**35**), 1,8-dihydroxy-3,4-dimethoxyxanthone 5-*O*-β-d-glucopyranoside (**36**), mangiferin (**37**), and homomangiferin (**38**) together with 22 iridoids reference compounds, gentiiridoside A (**39**), gentiiridoside B (**40**), secologanol (**41**), secologanin (**42**), secologanoside (**43**), secoxyloganin (**44**), (*E*)-aldosecologanin (**45**), 5α-carboxystricrosidine (**46**), sweroside (**47**), swertiapunimarin (**48**), deacetylcentapicrin (**49**), decentapicrin A (**50**), trifloroside (**51**), swertiamarin (**52**), eustomoside (**53**), gentiopicroside (**54**), 6′-*O*-β-d-glucopyranosyl gentiopicroside (**55**), loganic acid (**56**), loganin (**57**), 8-epiloganin (**58**), 7-ketologanin (**59**), and swertiaside (**60**) prepared by Liu [23] were used for UHPLC-Q Exactive-Orbitrap MS analysis; their purities were above 98%.

CC was carried out on Macroporous resin D101 (Haiguang Chemical Co., Ltd., Tianjin, China). Analytical pure ethanol (EtOH) and petroleum ether (PE) were purchased from Tianjin Concord Technology Co., Ltd. (Tianjin, China). Acetonitrile (ACN), methanol (MeOH), and formic acid (FA) of HPLC grade were supplied by Thermo Fisher, Waltham, MA, USA. Ultra-pure water was prepared with a Milli-Q purification system (Millipore, Burlington, MA, USA).

Penicillin, streptomycin, trypsin, dimethyl sulfoxide (DMSO), and MTT were ordered from Thermo Fisher Scientific, Inc., Waltham, MA, USA. Embryonic bovine serum, RPMI-1640 medium, and penicillin-streptomycin solution were provided by Viva Cell Bioscience (Shanghai ProPen Biotechnology Co., Ltd., Shanghai, China). Human CRC cell lines HT29 and LoVo were purchased from the Cell Resource Center, Shanghai Institutes for Biological Sciences of the Chinese Academy of Sciences (Shanghai, China).

### 4.2. Sample Preparation and Chemical Composition Analysis

The dried GA (1.5 kg) was cut into small pieces and successively extracted under reflux with 22.5 L 70% EtOH for 3 h, 2 h, and 2 h. A residue (TE, 357.0 g) was provided after removal of the solvent under reduced pressure. TE (321.3 g) was dissolved in 4 L of distilled H_2_O, then partitioned with PE (1:1, *v*/*v*) three times to obtain the PE layer (PF, 19.0 g) and the H_2_O layer (AF, 302.3 g). AF (300.0 g) was re-dissolved with 9 L of distilled H_2_O and centrifugated at 4000 rpm to obtain the supernatant and sediment (105.0 g). The supernatant was successively subjected to D101 macroporous resin CC [bed volume (BV) was 9.0 L] and gradient eluted with 4 BV H_2_O, 10% EtOH, 20% EtOH, and 95% EtOH. According to the LC-MS analysis result, the water fraction (WF, 58.0 g) eluted with 4 BV H_2_O, iridoids fraction (IF, 42.0 g) eluted with 4 BV 10% EtOH and 0–2.5 BV 20% EtOH were gained, respectively. Meanwhile, 2.5–4 BV 20% EtOH and 4 BV 95% EtOH eluates were combined with the sediment to obtain xanthones fraction (XF, 196.0 g).

TE, AF, WF, IF, and XF were prepared in MeOH at a final concentration of approximately 10 mg/mL and centrifuged at 14,000 rpm for 15 min, then the supernatant was added to the injection vials for analysis. Meanwhile, standard test solutions of the above-mentioned reference compounds (in Section 4.1) were prepared in MeOH at a final concentration of approximately 100 ng/mL. All stock solutions were stored at 4 °C in darkness and brought to room temperature before use.

Identification of TE, AF, WF, IF, and XF was performed on a Thermo UltiMate 3000 UHPLC combined with an ESI-Q-Orbitrap MS (Thermo, Waltham, MA, USA). Samples were separated on an Acquity UPLC HSS T3 (1.8 µm, 2.1 × 100 mm) column with a column temperature of 30 °C and a flow rate of 0.4 mL/min. A mobile phase composed of FA-H_2_O (1:1000, *v*/*v*) (A) and ACN (B) in the gradient program: 0–6 min, 15–45% B; 6–9 min, 45% B, 9–10 min, 95% B; 10–14 min, 95% B was used. An equilibration of 3 min was used between successive injections. An aliquot of 2 µL of each sample was injected for analysis.

The detection was performed in the negative ESI mode (capillary voltage: 3.2 kV). Ultra-high purity N_2_ and high purity N_2_ were used as the collision gas and the sheath/auxiliary gas, respectively. The ESI source parameters were 320 °C for capillary temperature, 300 °C for ion source heater temperature, 40 L/h for sheath gas (N_2_), and 10 L/h for auxiliary gas (N_2_), and the collision energy of the quadrupole ranging between 35 V were used. The mass range of the Orbitrap analyzer scanner was *m*/*z* 100 to 1500 in a full mass acquisition mode. Selected precursors analyzed more than two times were actively excluded from analysis for 10 s. Monitoring time was 0–14 min. Data recording and processing were performed using the Xcalibur 4.0 software (Thermo Fisher Scientific, Inc., Waltham, MA, USA). The accuracy error threshold was fixed at 5 ppm.

### 4.3. Cell Culture

Human CRC cell lines HT29 and LoVo were cultured in RPMI 1640 medium containing 10% fetal bovine serum and 1% penicillin-streptomycin at 37 °C in a humidified environment under 5% CO_2_. Until the cells were grown to 90% confluence, the related bioassays were performed.

### 4.4. Cell Proliferation Assay

The proliferation inhibitory activities of TE, PF, AF, WF, IF, XF, and 38 xanthones on HT29 and LoVo cells were assessed by the MTT method. When the cells were grown to 90% confluence, they were seeded in 96-well plates at a density of 1 × 10^4^ cells/well. After being incubated for 24 h, the cells were cultured in a medium with the sample at gradient concentrations of 500, 250, 125, 62.5, and 31.25 μg/mL for TE, PF, AF, WF, IF, and XF and 100, 50, 25, 12.5, and 6.25 μM for 38 xanthones for 48 h at 37 °C in an incubator with 5% CO_2_, respectively. Then, the medium was discarded and replaced by adding 100 μL of medium containing 500 μg/mL MTT per well. After 4 h of incubation, the culture medium was replaced by 100 μL DMSO. After shaking and mixing for 30 s, the absorbance was measured at 490 nm with a microplate spectrophotometer (Synergy TM NEO, BioTek Instruments, Winooski, VT, USA). The experiment was repeated at least three times and the mean and standard deviation were obtained using Graphpad prism 8.0 software.

### 4.5. Colony Formation Assay

HT29 and LoVo cells were cultured in 6-well plates at a density of 1 × 10^3^ cells/well for 24 h, and the medium containing 0, 20, 40, and 80 μg/mL XF was added. The cells were incubated continuously for 14 d at 37 °C in an incubator with 5% CO_2_. In the process, the solution was changed every 3 days. After 14 d of incubation, the medium was discarded, washed 3 times with PBS, and fixed with 4% paraformaldehyde at room temperature for 15 min; then, it was stained with 0.1% crystal violet for 30 min, washed again with PBS, air-dried, and photographed. The number of cell clones was calculated with Image J software and counted with Graphpad prism 8.0 software.

### 4.6. Scratch Wound Assay

HT29 and LoVo cells at a density of 5 × 10^6^ cells/mL were seeded on 6-well culture plates and incubated for 24 h, respectively. Upon attaining 80% confluency, the medium was discarded, and the wells were scratched with a 1 mL sterile micropipette tip. The cells were rinsed with PBS to remove the scratched cells, and the width of the scratch was recorded using an inverted microscope (0 h). After 48 h, administration of 0, 20, 40, and 80 μg/mL XF in RPMI 1640 medium at 37 °C and 5% CO_2_, the wound closure was viewed microscopically and photographed. The scratch area was calculated by Image J software and counted with Graphpad prism 8.0 software.

### 4.7. Cell Invasion Assay

A total of 60 μL Matrigel gel dilution was added to the upper chamber of the transwell and placed in the incubator at 37 °C in 5% CO_2_ overnight to form a hydration film. Then, 200 μL cell suspension at a density of 4 × 10^4^ cells/well was added to the upper chamber, and 600 μL of medium containing 0, 20, 40, and 80 μg/mL XF was added to the lower chamber. After 48 h of incubation, the chamber was washed three times with PBS, and the cells on the surface of the upper chamber were successively wiped off. The cells were fixed with 4% paraformaldehyde for 15 min and then stained with 0.1% crystal violet for 30 min. After drying, the cells were photographed under a microscope. The number of cells on the chamber membrane was counted using Image J and Graphpad Prism 8.0 software.

### 4.8. Detection of Apoptosis by Flow Cytometry

Cells were inoculated in six-well plates at 5 × 10^5^ cells/well and treated with XF (0, 20, 40, and 80 μg/mL) for 24 h. Cells were harvested and centrifuged at 300× *g* for 10 min, then measured by Annexin V-FITC/PI Apoptosis Detection kit under the guidance of the instructions. Samples were stained with 5 μL Annexin V-FITC for 10 min and with 5 μL PI for 5 min in the dark, and immediately evaluated by flow cytometry. The apoptosis rates were calculated by a sum of the total percentages of the cells in the right lower quadrant (early apoptosis) and the right upper quadrant (late apoptosis) [50].

### 4.9. Experiment of Network Pharmacology

#### 4.9.1. Prediction of Target Proteins

The SMILES format of the 38 xanthones obtained from GA was generated from Chemdraw. Then, the compound-related targets corresponding to them were gained through TargetNet [51] (prob value is greater than 0.5), STITCH [52] (tanimoto score is greater than 0.9), Swiss-Target-Prediction [53] (the score is greater than 0.4), and SEA [54] (the score is greater than 0.4) databases for target analysis and prediction by uploading their SMILES structures.

“Colorectal cancer” and “colorectal carcinoma” were used as keywords to search CRC-related targets from GeneCards [55] (relevance score is greater than 50), Drugbank [56] and DisGetNET [57] (prob value is greater than 0.3) databases. Then, the protein targets related to CRC and 38 xanthones obtained from the above databases were both pooled and entered into the Uniport database [58], with the restriction “Homo sapiens” to gain its related gene name, gene ID, and functions. The online Venn diagram [59] tool was employed for the identification of the overlapping targets of 38 xanthones and CRC, thus obtaining the candidate targets of 38 xanthones for CRC treatment.

#### 4.9.2. Construction of Protein-Protein Interaction (PPI) Network

The candidate targets of 38 xanthones for CRC treatment were loaded onto the Search Tool for the Retrieval of Interacting Genes (STRING) database [60] to acquire protein-protein interaction results. The results were imported into Cytoscape 3.7.1 software to visualize the PPI network. Then, the topological analysis of the PPI network was carried out. According to the calculated degree, betweenness centrality (BC), and closeness centrality (CC) parameters, the key intervention targets of 38 xanthones obtained from GA in the prevention and treatment of CRC were identified.

#### 4.9.3. Enrichment Analysis of GO Function and KEGG Pathway

The key intervention targets of 38 xanthones in the prevention and treatment of CRC were imported into Metascape [61] for Gene Ontology (GO) function and Kyoto Encyclopedia of Genes and Genomes (KEGG) pathway enrichment analysis. GO is evaluated by cellular components (CCs), molecular functions (MFs), and biological processes (BPs). More targets appearing in the KEGG pathway indicated a stronger correlation, suggesting that this pathway may be a potential mechanism of drug action on the disease. *p* < 0.05 was used as the threshold to screen the potential signaling pathway and anti-CRC mechanism of xanthones. In addition, the results of GO and KEGG analyses were plotted on the bioinformatics platform [62].

#### 4.9.4. Construction of Component-Target-Pathway Visualization Network

The component-disease predicted targets and target-pathway relationships were imported into Cytoscape 3.7.1 software to construct a component-target-pathway network to visualize and analyze the therapeutic mechanisms of key xanthones for CRC.

### 4.10. Molecular Docking Simulation

The ligand files of 1,3,5-trihydroxyxanthone (**16**), 1,3,5,8-tetrahydroxyxanthone (**17**), 1,5,8-trihydroxy-3-methoxyxanthone (**18**), 1,7-dihydroxy-3,8-dimethoxyxanthone (**19**), 1,7-dihydroxy-3,4-dimethoxyxanthone (**22**), and 1,7-dihydroxy-3,4,8-trimethoxyxanthone (**23**) in SDF format were downloaded from the PubChem database [63] and then energetically optimized in Chem3D 15.1 software to obtain the mol2 format files.

The RCSB PDB database [64] was used to download the receptor proteins, 2RD0 (phosphoinositide-3-kinase, PIK3CA), 2UZS (protein kinase Bα, Akt1), and 1AUE (mammalian target of rapamycin, mTOR). It should be noted that proteins with a resolution of less than 3.5 Å should be selected, and the main and side chains of proteins below 3.5 Å are generally clear enough to meet the requirements of molecular docking. Proteins are processed and modified by Pymol, AutoDock Vina, and MGLtools to meet docking requirements, such as removing solvent, and computer gasteiger.

AutoDock Vina is a computational docking program based on scoring functions and gradient-optimized conformational search [40,65]. After preparing the ligand and receptor, molecular docking was performed using the AutoDock Vina and MGLtools to predict the best binding mode between ligands **16**–**19**, **22**, and **23** and receptors PIK3CA, Akt1, and mTOR. The result of docking is judged by the affinity value, the lower the affinity value, the stronger the binding ability between ligand and receptor. Generally, the docking results are considered reliable when the affinity value is less than −5 kcal/mol. The binding sites were analyzed by the protein-ligand interaction profiler website [66]. Then, the binding site was visualized by Pymol software.

### 4.11. Western Blot Assay

HT29 and LoVo cells were homogenized with 10-folds of RIPA Lysis Buffer (adding 1 mmol/L PMSF; phosphatase inhibitor) under ice bath conditions, and the mixtures were centrifuged at 12,000× *g* for 10 min. The supernatants were collected, and the total protein concentration was determined by Cytation 5 (BioTek, Winooski, VT, USA), then the denatured protein samples were obtained after they were boiled at 100 °C for 10 min and were saved at −20 °C or −80 °C until use.

Samples were separated by 10% SDS-polyacrylamide gel electrophoresis and transferred electrophoretically onto a PVDF membrane (Merck Millipore, Bedford, MA, USA). Hereafter, they were incubated overnight individually at 4 °C with anti-phosphoinositide 3-kinase (anti-PI3K) (110α) (4249, Cell Signaling Technology, Danvers, MA, USA), anti-Akt (4685S, Cell Signaling Technology, Danvers, MA, USA), anti-Phospho-Akt (Ser473) (40060, Cell Signaling Technology, Danvers, MA, USA), anti-mTOR (2972, Cell Signaling Technology, Danvers, MA, USA), anti-Phospho-mTOR (Ser2448) (5536, Cell Signaling Technology, Danvers, MA, USA), anti-B cell lymphoma/lewkmia-2 (anti-Bcl2) (A0208, ABclonal Technology, Wuhan, China), anti-Bcl2-associated X protein (A19684, anti-Bax) (ABclonal Technology, Wuhan, China), anti-caspase 3 (A0214, ABclonal Technology, Wuhan, CN), anti-cleaved-caspase 3 (9661, Cell Signaling Technology, Danvers, MA, USA), and anti-β-actin (ab8227, Abcam plc., Cambridge, UK) antibodies diluted in Tris Buffered Saline with Tween 20 (PBST, Beijing Solarbio Science & Technology Co. Ltd., Beijing, China). Then, the blots were washed three times with PBST and incubated with the goat anti-rabbit IgG (Abcam plc., Cambridge, UK) conjugated with horseradish peroxidase at room temperature for 1 h. Subsequently, blots were washed three times with PBST and then mixed with Enhanced Chemiluminescence (Millipore Co., Ltd., Bedford, MA, USA). After that, the protein bands were visualized with the Amersham imager 600 System (GE Healthcare, Chicago, IL, USA). The band intensities were quantified using Image J analysis software. The relative protein expression level of each band was normalized by β-actin [67].

### 4.12. Statistical Analysis

Data are expressed as the mean ± S.E.M. Significant differences between means were evaluated by *t* test and one-way analysis of variance (ANOVA) using Graphpad prism 8.0 statistical software. *p* < 0.05 was considered to represent a statistically significant difference [68].

## 5. Conclusions

This study demonstrated xanthones 1,3,5-trihydroxyxanthone (**16**), 1,3,5,8-tetrahydroxyxanthone (**17**), 1,5,8-trihydroxy-3-methoxyxanthone (**18**), and 1,7-dihydroxy-3,8-dimethoxyxanthone (**19**) to be the effective compounds of GA inhibiting CRC. The mechanism may be related to the regulation of PI3K/Akt/mTOR signaling pathway transduction, at least. Importantly, it is necessary to conduct a more in-depth study of 1,3,5,8-tetrahydroxyxanthone (**17**), the most abundant active xanthone in GA, to provide more choices for the discovery of anti-CRC drugs.

## Figures and Tables

**Table 1:** IC_50_ values of component groups obtained from GA on HT29 and LoVo cells.

No.	IC_50_ (μg/mL)	
	HT29	LoVo
TE	386.63 ± 4.19	384.20 ± 6.80
PF	366.63 ± 6.42	303.50 ± 6.28
AF	321.67 ± 5.76	256.60 ± 5.32
WF	>500	>500
IF	>500	>500
XF	85.60 ± 2.49	76.30 ± 2.16

Values were means ± SEM of three replications.

**Table 2:** IC_50_ values of xanthones **1**–**38** on HT29 and LoVo cells.

No.	IC_50_ (μM)		No.	IC_50_ (μM)	
	HT29	LoVo		HT29	LoVo
**1**	>100	>100	**20**	>100	>100
**2**	>100	>100	**21**	>100	>100
**3**	>100	>100	**22**	45.86 ± 1.54	35.41 ± 1.45
**4**	>100	>100	**23**	55.79 ± 2.62	45.72 ± 2.14
**5**	>100	>100	**24**	>100	>100
**6**	>100	>100	**25**	>100	>100
**7**	>100	>100	**26**	>100	>100
**8**	>100	>100	**27**	>100	>100
**9**	>100	>100	**28**	>100	>100
**10**	>100	>100	**29**	>100	>100
**11**	>100	>100	**30**	>100	>100
**12**	>100	>100	**31**	>100	>100
**13**	>100	>100	**32**	>100	>100
**14**	>100	37.42 ± 0.70	**33**	>100	46.82 ± 2.79
**15**	>100	>100	**34**	>100	>100
**16**	64.31 ± 2.90	75.93 ± 2.73	**35**	88.65 ± 5.17	>100
**17**	83.42 ± 3.88	69.87 ± 3.71	**36**	>100	>100
**18**	90.21 ± 4.57	85.32 ± 3.05	**37**	>100	>100
**19**	87.93 ± 5.03	88.64 ± 4.32	**38**	>100	>100

Values were means ± SEM of three replications.

**Table 3:** Molecular docking parameter information.

Ligand (Compound)	Receptor PDB ID (Protein)	Affinity (kcal/mol)	Binding Site
**16**	2RD0 (PIK3CA)	−7.8	ASN-457, LEU-452, ILE-459, VAL-461, GLN1014, GLY-1007, GLY-1009
	2UZS (Akt1)	−6.4	TRP-11, PRO-24, GLU-95, HIS-89, HIS-13, GLU-91
	1AUE (mTOR)	−6.6	ILE-932, TYR-836, VAL-851, SER-854
**17**	2RD0 (PIK3CA)	−7.8	ARG-992, GLN-993, ASP-538, ARG-537
	2UZS (Akt1)	−6.2	LYS-20, LYS-17, ARG-15, THR-87, ARG-86, GLU-85
	1AUE (mTOR)	−6.2	GLN-2083, ALA-2057
**18**	2RD0 (PIK3CA)	−8.2	ARG-398, PRO-609, GLU-642, LEU-648
	2UZS (Akt1)	−5.6	SER-56, GLN-59, GLU-114,
	1AUE (mTOR)	−8.2	PHE-2019, TYR-2105, TYR-2106, LEU-2032, TRP-2102
**19**	2RD0 (PIK3CA)	−7.9	SER-854, PHE-930, VAL-851, ILE-932, TYR-836, ILE-848, LYS-802
	2UZS (Akt1)	−6.3	PRO-24, TRP-11, GLU-95, GLU-91
	1AUE (mTOR)	−8.0	PHE-2019, TYR-2106, LEU-2032, TRP-2102, PHE-2040, ASP-2103
**22**	2RD0 (PIK3CA)	−7.7	SER-1015, ILE-459, LEU-452
	2UZS (Akt1)	−5.9	LEU-110, VAL-106, THR-105
	1AUE (mTOR)	−7.7	PHE-2019, TYR-2106, TYR-2105, TRP-2102
**23**	2RD0 (PIK3CA)	−7.3	LEU-645, LYS-678, GLN-682
	2UZS (Akt1)	−5.6	TRP-424, TRP-446, PRO-447, THR-679, ASN-677, GLY-1009, HIS-450
	1AUE (mTOR)	−7.3	PHE-2040, SER-2036

## Data Availability

All data will be available upon request.

## Supplementary Materials

The following supporting information can be downloaded at: https://www.mdpi.com/article/10.3390/ijms24032279/s1.
