# Supplementary material for: Xanthones from Gentianella acuta (Michx.) Hulten Ameliorate Colorectal Carcinoma via the PI3K/Akt/mTOR Signaling Pathway
Source: Int J Mol Sci. 2023 Jan 23;24(3):2279. doi: 10.3390/ijms24032279 (PMC9917368; doi:10.3390/ijms24032279)
Supplement: Supplementary file 1 [file ijms-24-02279-s001.zip › ijms-2177590-supplementary.pdf]

Supplementary information

Table S1. The qualitative analysis of XF and IF by UPLC-ESI-Q-Orbitrap MS. ……………. 3
Table S2. The SMILES code of compounds in XF. ……………………………………………8
Table S3. Database about the targets of compound and disease. ……………………………. 20
Table S4. The major targets of xanthones for CRC treatment and the topological parameter. .. 22
Figure S1. BPC for the mixed references. ……………………………………………………… 24
Figure S2. The raw data of Fig. 3E. ……………………………………………………………… 25
Figure S3. The raw data of Fig. 7A. ……………………………………………………………… 26
Figure S4. The raw data of Fig. 7B. ……………………………………………………………… 27
Figure S5. The raw data of Fig. 7C. ……………………………………………………………… 28
Figure S6. The raw data of Fig. 7D. ……………………………………………………………… 29
Figure S7. The raw data of Fig. 7E. ……………………………………………………………… 30
Figure S8. The raw data of Fig. 7F. ……………………………………………………………… 31
Figure S9. The raw data of Fig. 8. ………………………………………………………………… 32

Table S1. The qualitative analysis of XF and IF by UPLC-ESI-Q-Orbitrap MS.

Peak No.	Compound Name	t_R (min)	Formula	Theoretical ESI-NI (*m*/*z*)	Measured ESI-NI (*m*/*z*)	Error (ppm)	Identification or Main fragment ions
**1**	2-Hydroxyethyl-3-carboxymethyl-5,7-dihydroxychromone	2.68	$C_{13}H_{12}O_7$	279.04993	279.05103	4.81	279.05103 (100)
**2**	Gentichromone A2	3.93	$C_{14}H_{14}O_7$	293.06558	293.06668	4.17	293.06668 (100), 278.04309 (11.21)
**3**	Gentichromone A3	1.22	$C_{19}H_{22}O_{12}$	441.10275	441.10385	3.27	441.10385 (100)
**4**	1,3,5,8-Tetrahydroxyxanthone 3-*O*-*β*-D-glucopyranoside	4.21	$C_{19}H_{18}O_{11}$	421.07654	421.07763	3.19	421.07763 (100), 259.02484 (2.97)
**5**	1,3,5,8-Tetrahydroxyxanthone 1-*O*-*β*-D-glucopyranosyl(1→ 6)-*β*-D-glucopyranoside	2.01	$C_{25}H_{28}O_{16}$	583.12936	583.13046	1.38	583.13046 (100), 259.02435 (8.48)
**6**	1,3,8-Trihydroxy-4-methoxyxanthone 5-*O*-*β*-D-glucopyranoside	4.55	$C_{20}H_{20}O_{12}$	451.08710	451.08820	3.23	451.08820 (100), 436.06448 (3.88),
**7**	(5*R*,8*S*)-1,3,5,8-Tetrahydroxy-5,6,7,8-tetrahydroxanthone	2.62	$C_{13}H_{12}O_6$	263.05501	263.05611	4.66	263.05611 (100)
**8**	(5*S*,8*S*)-1,3,5,8-Tetrahydroxy-5,6,7,8-tetrahydroxanthone	2.16	$C_{13}H_{12}O_6$	263.05501	263.05611	3.86	263.05611 (100)
**9**	(5*R*,8*S*)-1-*O*-*β*-D-Glucopyranosyl-1,3,8-trihydroxy-5,6,7,8-tetrahydroxanthone	1.09	$C_{19}H_{22}O_{11}$	425.10784	425.10893	4.90	425.10893 (100)
**10**	(5*R*,8*S*)-3-*O*-*β*-D-Glucopyranosyl-1,3,8-trihydroxy-5,6,7,8-tetrahydroxanthon	1.27	$C_{19}H_{22}O_{11}$	425.10784	425.10893	1.61	425.10893 (100)
**11**	(5*R*,8*S*)-8-*O*-*β*-D-Glucopyranosyl-1,3,8-t	2.05	$C_{19}H_{22}O_{11}$	425.10784	425.10893	3.84	425.10893 (100)

	rihydroxy-5,6,7,8-tetrahydroxanthone						
**12**	(5*R*,8*S*)-8-*O*-*β*-D-Xylopyranosyl-1,3,8-trihydroxy-5,6,7,8-tetrahydroxanthone	2.49	$C_{18}H_{20}O_{10}$	395.09727	395.09837	3.21	395.09837 (100), 259.02475 (5.57)
**13**	1-*O*-*β*-D-Glucopyranosyl-3,5,8-trihydroxyxanthone	7.27	$C_{19}H_{22}O_{11}$	425.10784	425.10893	3.53	425.10893 (100), 245.04536 (32.36)
**14**	Norswertianolin	1.41	$C_{19}H_{22}O_{11}$	425.10784	425.10893	4.76	425.10893 (100), 245.04536 (32.37)
**15**	Amarellin B	3.10	$C_{18}H_{20}O_{10}$	395.09727	395.09918	4.83	ND
**16**	1,3,5-Trihydroxyxanthone	6.05	$C_{13}H_{8}O_{5}$	243.02880	243.02990	4.98	243.02990 (100)
**17**	1,3,5,8-Tetrahydroxyxanthone	6.62	$C_{13}H_{8}O_{6}$	259.02371	259.02481	3.77	259.02481 (100)
**18**	1,5,8-Trihydroxy-3-methoxyxanthone	9.14	$C_{14}H_{10}O_{6}$	273.03936	273.04046	5.15	273.04046 (100)
**19**	1,7-Dihydroxy-3,8-dimethoxyxanthone	8.53	$C_{15}H_{12}O_{6}$	287.05501	287.05000	4.53	287.05000 (100)
**20**	1,3,8-Trihydroxy-4,5-dimethoxyxanthone	9.23	$C_{15}H_{12}O_{7}$	303.04993	303.05103	4.13	303.05103 (100), 259.04059 (21.45)
**21**	1,3,8-Trihydroxy-4,7-dimethoxyxanthone	7.90	$C_{15}H_{12}O_{7}$	303.04993	303.05103	5.22	303.05103 (100), 259.02505 (21.90)
**22**	1,7-Dihydroxy-3,4-dimethoxyxanthone	5.91	$C_{15}H_{12}O_{6}$	287.05501	287.05611	3.12	287.05611 (100), 272.03284 (23.74)
**23**	1,7-Dihydroxy-3,4,8-trimethoxyxanthone	7.23	$C_{16}H_{14}O_{7}$	317.06558	317.06668	4.92	311.06668 (100), 259.02521 (47.46), 174.95555 (40.48)
**24**	1-*O*-*β*-D-Glucopyranosyl-3,5,8-trihydroxyxanthone	2.56	$C_{19}H_{18}O_{11}$	421.07654	421.07763	3.47	421.07763 (100)
**25**	1,3,8-Trihydroxyxanthone-5-*O*-*β*-D-glucopyranoside	4.46	$C_{19}H_{18}O_{11}$	421.07654	421.08546	4.37	421.08546 (100)
**26**	8-*O*-*β*-D-Glucopyranosyl-1,3,5,8-trihydroxyxanthone	2.39	$C_{19}H_{18}O_{11}$	421.07654	421.07763	3.12	421.07763 (100)

**27**	Swertianolin	3.77	$C_{20}H_{20}O_{11}$	435.09219	435.09328	2.67	435.09328 (100), 338.98148 (9.75)
**28**	Triptexanthoside A	3.84	$C_{19}H_{18}O_{11}$	421.07654	421.07763	2.66	421.07763
**29**	3,8-Dimethoxy-7-hydroxyxanthone 1-*O*-*β*-D-glucopyranoside	8.49	$C_{21}H_{22}O_{11}$	449.10784	449.05893	4.67	449.05893 (100), 259.02518 (19.90)
**30**	1-*O*-[*β*-D-Xylopyranosyl(1→ 6)-*β*-D-glucopranosyl]-7-hydroxyl-3,8-d imethoxyxanthone	3.72	$C_{26}H_{30}O_{15}$	581.15010	581.15119	2.06	581.15119 (100), 465.10477 (12.03)
**31**	3,7,8-Trimethoxyxanthone 1-*O*-*β*-D-glucopyranoside	4.14	$C_{22}H_{24}O_{11}$	463.12349	463.12458	3.48	463.12458 (100), 371.13492 (12.82), 160.84119 (5.23)
**32**	1-*O*-[*β*-D-Xylopyranosyl(1→ 6)-*β*-D-glucopranosyl]-3,7,8-trimethoxy xanthone	4.63	$C_{27}H_{32}O_{15}$	595.16575	595.06232	2.83	595.06232 (100)
**33**	1-Hydroxy-3,4-dimethoxyxanthone 7-*O*-*β*-D-glucopyranoside	8.12	$C_{21}H_{22}O_{11}$	449.10784	449.10893	1.70	449.10893 (100), 381.22421 (10.29), 300.27142 (7.53)
**34**	3,8-Dihydroxy-4,5-dimethoxyxanthone 1-*O*-*β*-D-glucopyranoside	4.64	$C_{21}H_{22}O_{12}$	465.10275	465.10385	3.35	465.10385 (100), 303.05115 (79.80)
**35**	1-*O*-[*β*-D-Glucopyranosyl(1→ 6)-*β*-D-glucopyranosyl]-3,8-dihydroxy- 4,5-dimethoxyxanthone	3.48	$C_{27}H_{32}O_{17}$	627.15558	627.15667	1.87	627.15667 (100), 303.06069 (3.50)
**36**	1,8-Dihydroxy-3,4-dimethoxyxanthone 5-*O*-*β*-D-glucopyranoside	2.45	$C_{21}H_{22}O_{12}$	466.11058	467.09951	3.76	467.09951 (100), 423.07458 (3.23), 406.80737 (2.56), 259.94135 (2.06)
**37**	Mangiferin	1.76	$C_{19}H_{18}O_{11}$	421.07654	421.07763	3.19	421.07763 (100), 259.02484 (2.97)

**38**	Homomangiferin	2.03	$C_{20}H_{20}O_{11}$	435.09219	435.09354	3.11	435.09354 (100), 315.05118 (10.20), 273.0394 (14.04)
**39**	Gentiiridoside A	4.29	$C_{36}H_{42}O_{19}$	777.22366	777.22559	3.19	777.22559 (100)
**40**	Gentiiridoside B	0.69	$C_{28}H_{40}O_{19}$	679.20801	679.21638	3.53	679.21638 (100), 523.13098 (60.68), 203.01912 (81.60), 443.09656 (62.93),
**41**	Secologanol	2.15	$C_{17}H_{26}O_{10}$	389.14422	389.03903	3.20	151.03903 (100), 165.05457 (30.07), 177.05461 (82.04), 227.12532 (53.98), 389.17783 (37.84)
**42**	Secologanin	1.98	$C_{17}H_{24}O_{11}$	403.12349	403.12473	3.08	403.12473 (100), 393.09625 (14.05)
**43**	Secologanoside	1.26	$C_{16}H_{22}O_{11}$	389.10784	389.10977	4.97	389.10977 (100), 375.13031 (15.35)
**44**	Secoxyloganin	2.27	$C_{17}H_{24}O_{11}$	403.12349	403.12473	3.08	403.12473 (100), 393.09625 (14.05)
**45**	(*E*)-Aldosecologanin	3.45	$C_{34}H_{46}O_{19}$	757.25496	757.25605	4.18	757.25605 (100), 318.21191 (18.66)
**46**	5*α*-Carboxystricrosidine	2.97	$C_{28}H_{34}N_2O_{11}$	573.20789	573.23196	1.26	573.23196 (100), 534.05170 (11.90), 181.01283 (10.47)
**47**	Sweroside	1.97	$C_{16}H_{22}O_9$	357.11801	357.11911	4.74	357.11911 (48.59), 210.07576 (20.48), 197.08070 (39.00), 177.05455 (100)
**48**	Swertiapunimarin	1.35	$C_{22}H_{32}O_{14}$	519.17083	519.17390	4.52	519.17390 (100), 421.07834 (47.73), 289.16605 (34.79)
**49**	Deacetylcentapicrin	3.23	$C_{23}H_{26}O_{11}$	477.13914	477.14023	3.68	477.14023 (100), 421.07834 (47.73), 289.16605 (34.79)
**50**	Decentapicrin A	3.71	$C_{23}H_{26}O_{11}$	477.13914	477.13071	3.68	473.13113 (71.97), 421.0787 (100), 289.16632 (55.82)
**51**	Trifloroside	6.07	$C_{35}H_{42}O_{20}$	781.21857	–	–	ND

**52**	Swertiamarin	3.55	$C_{16}H_{22}O_{10}$	373.11292	373.11402	3.48	373.114021628054.5281.04346202 6375.9
**53**	Eustomoside	3.91	$C_{16}H_{22}O_{11}$	389.10784	389.10893	4.27	389.10893 (37.84), 227.12532 (53.98), 177.05461 (82.040, 151.03903 (100),
**54**	Gentiopicroside	1.94	$C_{16}H_{20}O_{9}$	355.10236	355.10346	4.60	355.08331 (63.92), 160.83856 (64.41)
**55**	6'-*O*-*β*-D-Glucopyranosyl gentiopicroside	1.70	$C_{22}H_{30}O_{14}$	517.15518	517.15628	2.15	517.15628 (30.30), 403.12610 (100), 305.10767 (45.40), 160.84169 (27.84)
**56**	Loganic acid	0.99	$C_{16}H_{24}O_{10}$	375.12857	375.12967	2.92	375.12967 (100), 213.07634 (45.72), 169.0860 (65.39)
**57**	Loganin	1.82	$C_{17}H_{26}O_{10}$	389.14422	389.14532	3.40	389.14532 (100), 345.11914 (18.91), 209.04495 (9.70), 121.06462 (10.77)
**58**	8-Epiloganin	1.28	$C_{17}H_{26}O_{10}$	389.14422	389.14532	3.40	389.10916 (100), 345.11914 (18.91), 209.04495 (9.70)
**59**	7-Ketologanin	1.89	$C_{17}H_{24}O_{10}$	387.12857	387.12967	4.90	371.13571 (75.25), 331.01053 (75.55), 160.84161 (100)
**60**	Swertiaside	3.50	$C_{23}H_{28}O_{12}$	495.14970	495.15039	1.39	ND

*The compounds unambiguously identified with the reference standards comparison

Table S2. The SMILES code of compounds in XF.

Peak No.	Compound Name	Compound structure	Canonical SMILES Structural Formulae
**1**	2-Hydroxyethyl-3-carboxymethyl-5,7-dihydroxychromone.	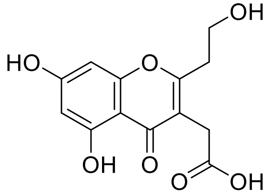	O=C1C2=C(C=C(O)C=C2O)OC(CCO)=C1CC(O)=O
**2**	Gentichromone A2	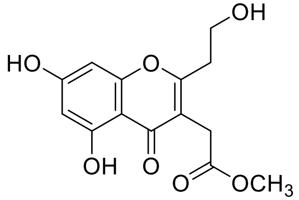	O=C1C2=C(C=C(O)C=C2O)OC(CCO)=C1CC(OC)=O
**3**	Gentichromone A3	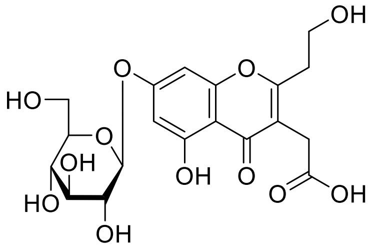	O[C@@H]1[C@@H](O)[C@H](O)[C@@H](CO)O[C@H]1OC(C=C2O)=CC3=C2C(C(CC(O)=O)=C(CCO)O3)=O

**4**	1,3,5,8-Tetrahydroxyxanthone 3-*O*-*β*-D-glucopyranoside	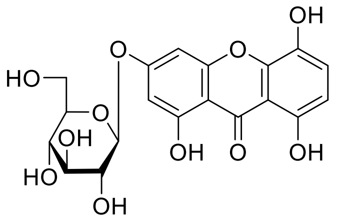	O=C1C2=C(C=C(O[C@H]3[C@H](O)[C@@H](O)[C@H](O)[C@@H](CO)O3)C=C2O)OC4=C1C(O)=CC=C4O
**5**	1,3,5,8-Tetrahydroxyxanthone 1-*O*-*β*-D-glucopyranosyl(1→6)-*β*-D-glucopyranoside	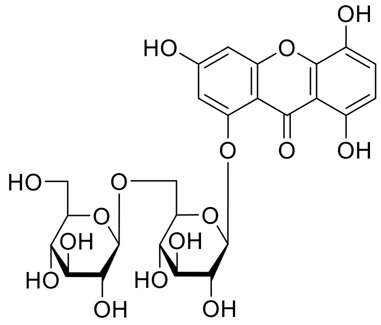	O=C1C2=C(C=C(O)C=C2O[C@H]3[C@H](O)[C@@H](O)[C@H](O)[C@@H](CO[C@H]4[C@H](O)[C@@H](O)[C@H](O)[C@@H](CO)O4)O3)OC5=C1C(O)=CC=C5O
**6**	1,3,8-Trihydroxy-4-methoxyxanthone 5-*O*-*β*-D-glucopyranoside	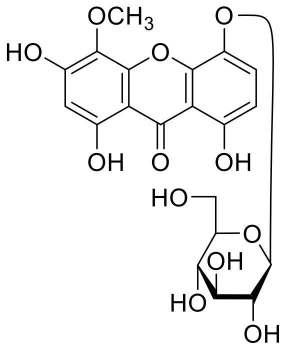	O=C1C2=C(C(OC)=C(O)C=C2O)OC3=C1C(O)=CC=C3O[C@@H]4[C@H](O)[C@@H](O)[C@H](O)[C@@H](CO)O4

**7**	(5*R*,8*S*)-1,3,5,8-Tetrahydroxy-5,6,7,8-tetrahydroxanthone	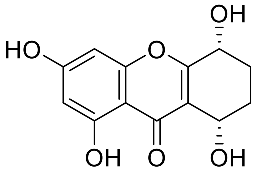	O=C1C2=C(C=C(O)C=C2O)OC3=C1C(O)CCC3O
**8**	(5*S*,8*S*)-1,3,5,8-Tetrahydroxy-5,6,7,8-tetrahydroxanthone	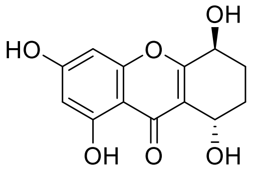	O=C1C2=C(C=C(O)C=C2O)OC3=C1C(O)CCC3O
**9**	(5*R*,8*S*)-1-*O*-*β*-D-Glucopyranosyl-1,3,8-trihydroxy-5,6,7,8-tetrahydroxanthone	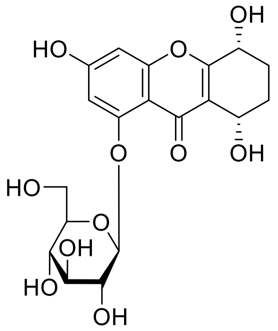	O=C1C2=C(C=C(O)C=C2O[C@H]3[C@H](O)[C@@H](O)[C@H](O)[C@@H](CO)O3)OC4=C1C(O)CCC4O
**10**	(5*R*,8*S*)-3-*O*-*β*-D-Glucopyranosyl-1,3,8-trihydroxy-5,6,7,8-tetrahydroxanthon	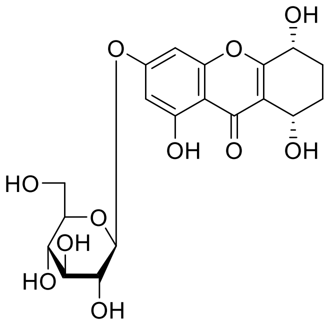	O=C1C2=C(C=C(O[C@H]3[C@H](O)[C@@H](O)[C@H](O)[C@@H](CO)O3)C=C2O)OC4=C1C(O)CCC4O

**11**	(5*R*,8*S*)-8-*O*-*β*-D-Glucopyranosyl-1,3,8-trihydroxy-5,6,7,8-tetrahydroxanthone	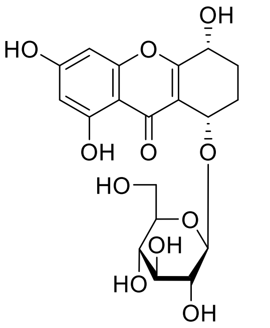	O=C1C2=C(C=C(O)C=C2O)OC3=C1C(O[C@H]4[C@H](O)[C@@H](O)[C@H](O)[C@@H](CO)O4)CCC3O
**12**	(5*R*,8*S*)-8-*O*-*β*-D-Xylopyranosyl-1,3,8-trihydroxy-5,6,7,8-tetrahydroxanthone	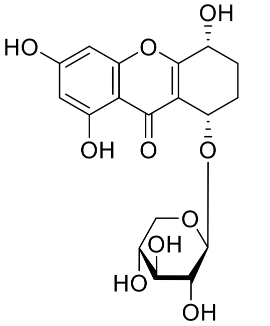	O=C1C2=C(C=C(O)C=C2O)OC3=C1C(O[C@H]4[C@H](O)[C@@H](O)[C@H](O)CO4)CCC3O
**13**	1-*O*-*β*-D-Glucopyranosyl-3,5,8-trihydroxyxanthone	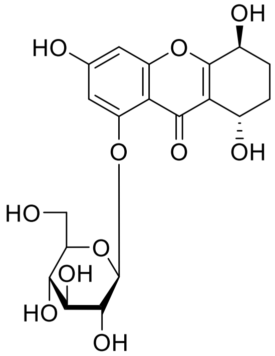	O=C1C2=C(C=C(O)C=C2O[C@H]3[C@H](O)[C@@H](O)[C@H](O)[C@@H](CO)O3)OC4=C1C(O)CCC4O

**14**	Norswertianolin	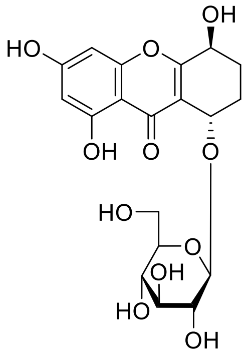	O=C1C2=C(C=C(O)C=C2O)OC3=C1C(O[C@H]4[C@H](O)[C@@H](O)[C@H](O)[C@@H](CO)O4)CCC3O
**15**	Amarellin B	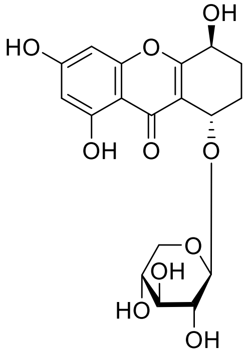	O=C1C2=C(C=C(O)C=C2O)OC3=C1C(O[C@H]4[C@H](O)[C@@H](O)[C@H](O)CO4)CCC3O
**16**	1,3,5-Trihydroxyxanthone	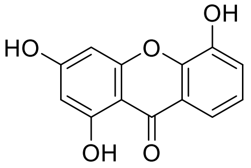	O=C1C2=C(C=C(O)C=C2O)OC3=C1C=CC=C3O

**17**	1,3,5,8-Tetrahydroxyxanthone	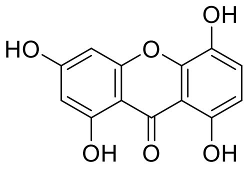	O=C1C2=C(C=C(O)C=C2O)OC3=C1C(O)=CC=C3O
**18**	1,5,8-Trihydroxy-3-methoxyxanthone	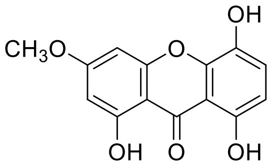	O=C1C2=C(C=C(OC)C=C2O)OC3=C1C(O)=CC=C3O
**19**	1,7-Dihydroxy-3,8-dimethoxyxanthone	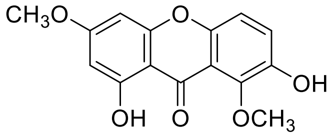	O=C1C2=C(C=C(OC)C=C2O)OC3=C1C(OC)=C(O)C=C3
**20**	1,3,8-Trihydroxy-4,5-dimethoxyxanthone	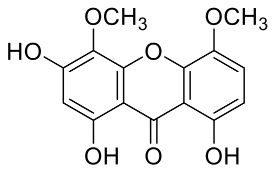	O=C1C2=C(C(OC)=C(O)C=C2O)OC3=C1C(O)=CC=C3OC
**21**	1,3,8-Trihydroxy-4,7-dimethoxyxanthone	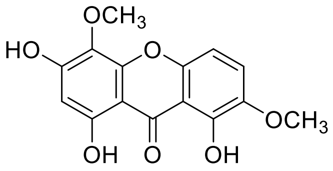	O=C1C2=C(C(OC)=C(O)C=C2O)OC3=C1C(O)=C(OC)C=C3

**22**	1,7-Dihydroxy-3,4-dimethoxyxanthone	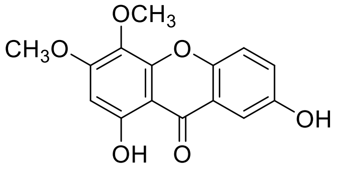	O=C1C2=C(C(OC)=C(OC)C=C2O)OC3=C1C=C(O)C=C3
**23**	1,7-Dihydroxy-3,4,8-trimethoxyxanthone	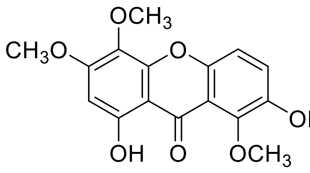	O=C1C2=C(C(OC)=C(OC)C=C2O)OC3=C1C(OC)=C(O)C=C3
**24**	1-*O*-*β*-D-Glucopyranosyl-3,5,8-trihydroxyxanthone	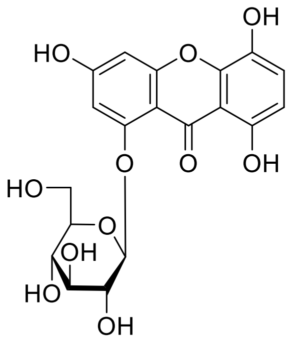	O=C1C2=C(C=C(O)C=C2O[C@H]3[C@H](O)[C@@H](O)[C@H](O)[C@@H](CO)O3)OC4=C1C(O)=CC=C4O

**25**	1,3,8-Trihydroxyxanthone-5-*O*-*β*-D-glucopyranoside	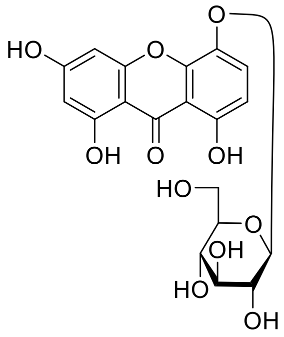	O=C1C2=C(C=C(O)C=C2O)OC3=C1C(O)=CC=C3O[C@@H]4[C@H](O)[C@@H](O)[C@H](O)[C@@H](CO)O4
**26**	8-*O*-*β*-D-Glucopyranosyl-1,3,5,8-trihydroxyxanthone	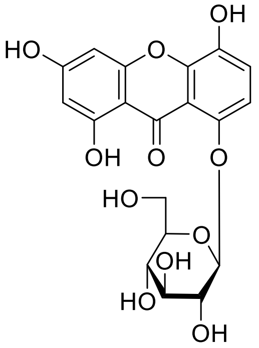	O=C1C2=C(C=C(O)C=C2O)OC3=C1C(O[C@H]4[C@H](O)[C@@H](O)[C@H](O)[C@@H](CO)O4)=CC=C3O

**27**	Swertianolin	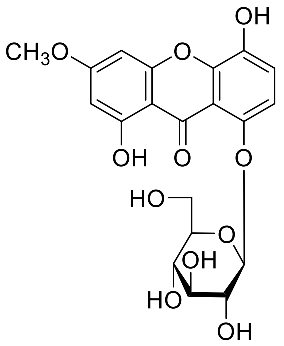	O=C1C2=C(C=C(OC)C=C2O)OC3=C1C(O[C@H]4[C@H](O)[C@@H](O)[C@H](O)[C@@H](CO)O4)=CC=C3O
**28**	Triptexanthoside A	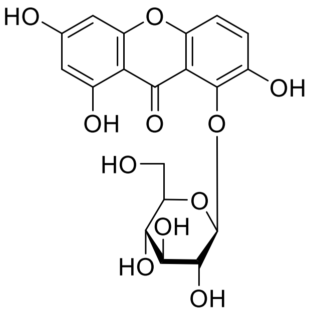	O=C1C2=C(C=C(O)C=C2O)OC3=C1C(O[C@H]4[C@H](O)[C@@H](O)[C@H](O)[C@@H](CO)O4)=C(O)C=C3
**29**	3,8-Dimethoxy-7-hydroxyxanthone 1-*O*-*β*-D-glucopyranoside	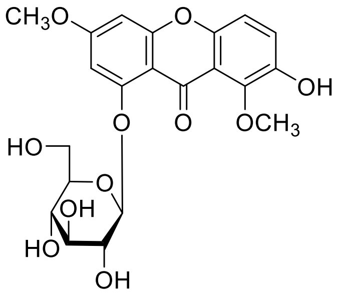	O=C1C2=C(C=C(OC)C=C2O[C@H]3[C@H](O)[C@@H](O)[C@H](O)[C@@H](CO)O3)OC4=C1C(OC)=C(O)C=C4

**30**	1-*O*-[β-D-Xylopyranosyl(1→6)-β-D-glucopranosyl]-7-hydroxyl-3,8-dimethoxyxanthone	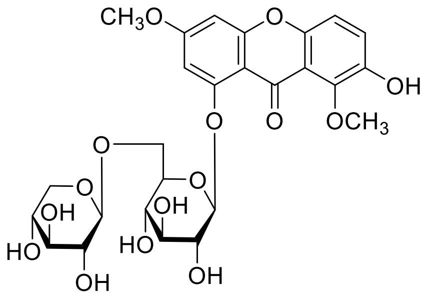	O=C1C2=C(C=C(OC)C=C2O[C@H]3[C@H](O)[C@@H](O)[C@H](O)[C@@H](CO[C@H]4[C@H](O)[C@@H](O)[C@H](O)CO4)O3)OC5=C1C(OC)=C(O)C=C5
**31**	3,7,8-Trimethoxyxanthone 1-*O*-β-D-glucopyranoside	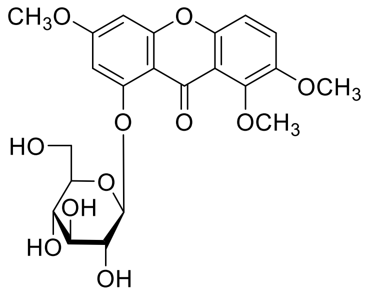	O=C1C2=C(C=C(OC)C=C2O[C@H](O3)[C@H](O)[C@@H](O)[C@H](O)[C@H]3CO)OC4=C1C(OC)=C(OC)C=C4
**32**	1-*O*-[β-D-Xylopyranosyl(1→6)-β-D-glucopranosyl]-3,7,8-trimethoxyxanthone	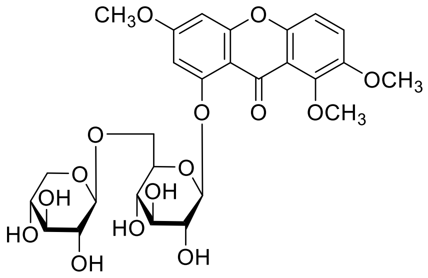	O=C1C2=C(C=C(OC)C=C2O[C@H]3[C@H](O)[C@@H](O)[C@H](O)[C@@H](CO[C@H]4[C@H](O)[C@@H](O)[C@H](O)CO4)O3)OC5=C1C(OC)=C(O)C=C5

**33**	1-Hydroxy-3,4-dimethoxyxanthone 7-*O*-*β*-D-glucopyranoside	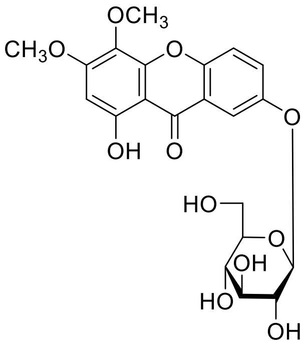	O=C1C2=C(C(OC)=C(OC)C=C2O)OC3=C1C=C(O[C@H]4[C@H](O)[C@@H](O)[C@H](O)[C@@H](CO)O4)C=C3
**34**	3,8-Dihydroxy-4,5-dimethoxyxanthone 1-*O*-*β*-D-glucopyranoside	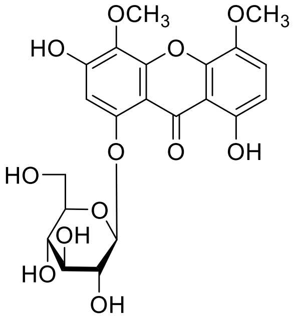	O=C1C2=C(C(OC)=C(O)C=C2O[C@H]3[C@H](O)[C@@H](O)[C@H](O)[C@@H](CO)O3)OC4=C1C(O)=CC=C4OC
**35**	1-*O*-[*β*-D-Glucopyranosyl(1→6)-*β*-D-glucopyranosyl]-3,8-dihydroxy-4,5-dimethoxyxanthone	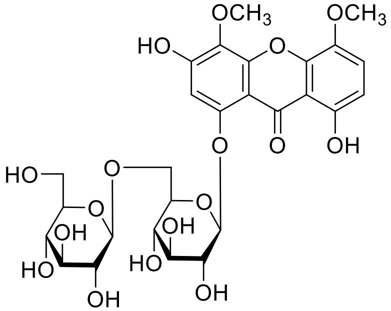	O=C1C2=C(C(OC)=C(O)C=C2O[C@H]3[C@H](O)[C@@H](O)[C@H](O)[C@@H](CO[C@H]4[C@H](O)[C@@H](O)[C@H](O)[C@@H](CO)O4)O3)OC5=C1C(O)=CC=C5OC

**36**	1,8-Dihydroxy-3,4-dimethoxyx anthone 5-*O*-*β*-D-glucopyranoside	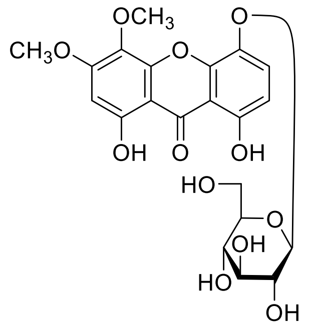	O=C1C2=C(C(OC)=C(OC)C=C2O)OC3=C1C(O)=CC=C3O [C@@H]4[C@H](O)[C@@H](O)[C@H](O)[C@@H](CO)O4
**37**	Mangiferin	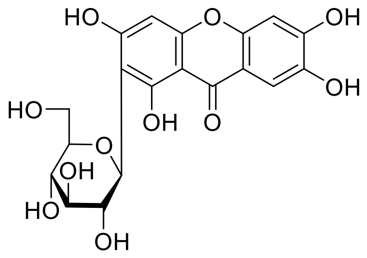	O=C1C2=C(C=C(O)C([C@H]3[C@H](O)[C@@H](O)[C@H] (O)[C@@H](CO)O3)=C2O)OC4=C1C=C(O)C(O)=C4
**38**	Homomangiferin	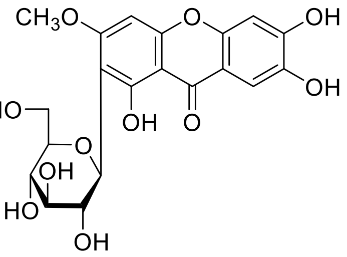	O=C1C2=C(C=C(OC)C([C@H]3[C@H](O)[C@@H](O)[C@ H](O)[C@@H](CO)O3)=C2O)OC4=C1C=C(O)C(O)=C4

Table S3. Database about the targets of compound and disease.

Groups	Total	Intersection of targets
Compound targets	294	ACE CAH4 ROCK1 ZAP70 SLC6A2 XDH TPSAB1 PRSS3P2 ADRA2C TTHY HSD17B1 RXRG HSD17B2 RAC1 ABCG2 PRKCG CA13 HTR1A CCNB1 RARA SLC6A4 PPBI PTPN1 CHRM5 CRHR1 MTNR1B AXL PPARA GRM2 CNR2 PPBN MGLL CP1B1 RORA ALDR ALOX5 DUSP3 HRH3 MAOB MAPKAPK2 PTGER2 CYP1A2 AOFA PTPN22 HDAC4 LYN CYP2D6 S1PR2 CYP2C19 NOS1 CA14 CA4 ROCK2 HSD17B3 ELANE TEK PLA2G1B ADRA2B PTGDR CES2 PLAT PIK3CG PLK1 HTR2C GALR3 IKBKB DPP4 TYK2 HTR7 DRD5 S1PR1 FOLH1 CXCR3 HRH4 MAOA ALDH2 LGUL CREB1 TYDP1 ATP4A DCOR CDK1 PNMT KCNA3 CHRNA4 PREP KIF11 HCRTR2 CP2C8 DRD3 CATL2 BDKRB1 PIK3CB THRB ADRA2A ADORA2A MAPK8 EDNRA AVPR1A HDAC8 GPR35 F10 CALCRL DGAT2 TNNI3 ADORA3 GNRHR OXTR CDC25C ADK CYP11B2 MRP1 HDAC2 PDE7A CHRFAM7A AKR1B1 CTSL CA2 PDIA1 HSP90AB1 PTGES ERAP1 OPRK1 CHRM1 HNF4A ALOX15 TBXA2R CACNA1B DRD4 CTDSP1 CDK5R1 SC5A2 TNNC1 ERR2 NOS3 ANPEP CCR3 CYP11B1 CETP KCNA5 SRD5A2 PTPN2 RIPK2 PDE10A METAP2 S28A3 PTGFR PKN1 CDK2 CNR1 AHR PDE4B PLIN5 CHRM2 PIM1 ADORA2B NEK6 ERR1 NR3C1 THRA HDAC3 BTK DPP8 FAAH CYP17A1 AL1B1 CHRM4 NUAK1 AL1A2 CYP19A1 CYP2C8 RARG STS MAPK14 HRH2 CAH13 SOX9 PTAFR ADRB1 CTSS CTSB SC5A1 TNKS2 LCK SIRT2 FURIN CASP9 CMA1 CACNA1H FYN HCRTR1 NMUR2 KCND3 CDK5 DRD2 CA5B LOX12 PAK4 HTR1E S1PR5 CAH9 FNTB EDNRB DPP7 GRIN2B ELAV1 CAH2 S1PR4 APOBEC3G SLC5A1 CAH12 TNNT2 F2 TYRO HCAR2 TBXAS1 CYP2B6 TAAR1 APOBEC3A HDAC1 GRM 4 3MG SLC5A2 MIF SIRT1 SC5A4 CYP2C9 NOX4 CHRM3 CA5A CTSK NPY1R HSD11B1 AKR1C3 LOX5 AGTR1 PIM3 PTGS1 AOFB HTR5A HTR2A PDE4D PDE3A AK1BA MDR1 TNKS1 MME CA1 PTGIR CA6 AGTR2 SOAT1 FFAR1 ACHE GSK3B FDPS DAPK1 SIGMAR1 MTNR1A MCL1 PERM CES1 CA12 GHSR CYP3A4 LOX15 CAH7 PDE4A EPHX2 SCN5A PTPN7 APP ALPL CACNA1G RPS6KA3 PTGER4 NR2F2 BCL2A1 SLC6A3 LTB4R HDAC6 LTA4H HSP90AA1 CHRNA7 CD5R1 CA7 CBR1 CAH1 TOP1 PLIN1 CCR4 DRD1 CLK1 KDM4E
Disease targets	562	VDR ABCA3 CD44 MMP2 DONSON ERBB4 ABCD3 HSPB1 ABCC6 COL3A1 RTN4 FASLG MIR126 MIR31 NBN IRS4 CXCR4 MIR26B CUBN OTOP2 ADAMTS18 IGF1 SOS1 MIR146A TGFB2 MIR20A WNT1 CTNND2 CEBPA-DT PPP1R14A PTEN TPX2 AXIN1 MMP7 EP300 PDGFRL IMD16 HNF1A STARD8 MIR195 SETD2 TRPM7 SLC11A2 FLT1 MIR451A SERPINB5 LGR6 ZEB1 SFRP5 SOD2 MIR205 SMAD2 FHL3 NXN MSH3 PRKCZ DPYSL2 MIR145 NR4A2 SLCO1B3 PTPRD BRCA2 MIR200B MCRS1 CHD1 FADS1 FLCN NAMPT GPNMB ABCA10 MIR34A AKR1B10 BRCA1 CTNNA1 SFXN1 MIR222 PTPN12 TGM2 NME2 DMKN CDO1 GSTM1 IL10 BMPR1A CDC14A TIMP3 ABCD4 MAPK1 ABCG1 ACACA IGF2 MIR18A PAIP2 MIR214 NFATC1 LRP1 POSTN CDH22 MTHFR BRIP1 SMAD3 BIRC5 PALB2 GNAS SDF4 FBN2 GUCY1A2 ABCA6 EGFR MIR1273C LIFR MTRR DICER1 ADAMTS15 TNFRSF9 MIR19A AXIN2 IFNA2 MIR200A PLA2G2A BOLL EZH2 CTLA4 MSH6 ST8SIA4 ERBB3 FAS SRSF6 MIR127 ERBB2 SLCO2A1 ARNT DDIT3 CEACAM5 CDKN1B NTHL1 CDH1 SLC22A15 MIR192 IFNG ATAD1 MIR34B ABCA1 MIR223 MIR200C TNFSF10 NAT2 FOXL2 MIR193A MUC1 CAD MIR142 KL KRT20 AKAP12 MIR27A UHRF2 CXCL12 RHEB CENPH MIR93 TE T2 ABCB10 ING4 MIR215 AMZ2P1 ADAM19 RRM2 MRE11 DCLK1 GUCY2C KLF5 AKAP9 RASSF2 NCR1 IPP FBLN2 FEN1 PRKD1 CYP1A1 ABCC5 TNS4 PIK3R2 OX40 MIR143 SMAD9 TXNRD1 DPYD APOB CASP8 TSC2 KDM2A QKI KDM6A MIR1271 LRP2 IL6ST MTDH MIR203A FGFR3 FLNC MIR34C RAD50 KRAS SYNE1 MTHFD1L GATA5 MIR181A1 CDX2 ABCB4 GTF2B MIR100 COL7A1 HRH1 PPP2R1B CHGA CHEK2 PKHD1 APDS TNFRSF10B MUTYH PAX8 NEURL1 KRT18 NT5C HIF1A TP73 MIR155 SELENOP MCC UNG TGFA GSTP1 FADS2 PROM1 MIR10B ZNF569 GNB4 TRIM28 CDC73 RB1 CFTR GSTM3 NRAS CXCL8 ZNF432 IMD14 WRN OGG1 TTLL3 SLC29A1 MSH2 ZEB2 CPAMD8 TIAM1 AFP FBXW7 NTNG1 MIR182 FN1

		HK3 BUB1B ABCC4 ZFP36L2 DHFR MT-CO1 SLC12A5 ODC1 KRT7 ERCC6 NFE2L2 ABCD2 DPEP1 ABCA4 TXGP1L POLE ABCA9 BRF1 ASAP3 TCERG1L MIR29C MIR22 ACIN1 TLR4 KRT19 ATP7B GNB5 BMP6 ABCA8 TCF3 TP53TG1 TENM1 RNF182 LEF1 MKI67 TNFRSF4 MIR17 CABYR VWA2 TP53 SATB2 MLH3 PDGFRB CDH7 ODAM MIR140 UMPS STK11 TNF CHL1 TGFBR2 MUC2 RAF1 VTI1A IFNA1 HNF1B EGF HHIP CD248 MEG3 PDGFD RAD51 RAD51D ERCC1 RASAL2 CDKN1A SMAD7 VEGFC MAP2K2 KLF2 ABCC2 MPO TCF7L2 PGM1 VIM CSMD3 F5 FGFR2 FAT1 XRCC3 PCNA ABCE1 KIT ATG5 NRCAM EPHA7 IMD38 ZMIZ1 CCND2 FHIT PPOX MIR106B LGR5 JAK2 PIK3R1 CACUL1 MAF CD226 PTPRJ STAT3 RAD54B CAPN10 GHRHR RHPN2 ABCB6 MIR130B MIR150 XAF1 EPHB6 GLI1 SPP1 DLC1 BARD1 FOXH1 CTBP1 ABCB11 XIAP WNT5A COX1 IDH1 PHETA1 BOC PER1 CUX1 EYA4 NF1 YAP1 MUC5AC TMEM238L RAD51C PRPS1 CTNNB1 MIR221 MAP2K1 H19 NFKB1 MIR183 LDB1 SOX17 ABCC1 MIR141 TGFBR1 TIMP2 ZNF217 IL1B ZKSCAN3 CNPPD1 TBX1 CPE GALNT12 RUNX1T1 CCND1 IGF2R PTPRU POLD1 CD93 PMS1 ANP32A ATM RASL11A GPX4 SFRP4 ABCC13 SPARC NFKBIA CBX5 WIF1 IGFBP3 ABCB5 TYMS OBSCN STRAP DCC GSE1 HOXD9 CALCOCO2 CD9 MLH1 POLD3 CSE1L HRAS CD46 PTPRT RNF43 ACSL5 GAPDH APC SMAD4 CDKN2B LINC00673 TLR2 VEGFA XRCC1 LAMA1 FZD4 SETBP1 WNK1 CDKN2A PPM1E LPAR1 TGFB1 ABCA5 GLI3 PTCH1 PMM2 AMACR GRIN2A ABCC8 DPAGT1 TFRC RASGRF2 BCL10 GREM1 VPS13A TFPI2 BRINP1 MYC SH3TC1 LIG3 ETV1 JUN SULT2B1 ADAMTSL3 TCN2 TIMP1 EPHB2 AR ABCA12 SESN2 CDKN3 PCSK2 NME1 MGMT BUB1 JPH3 HOXD1 NKX2-1 MIR483 SCG5 HDC BRAF TWIST1 MIR21 EIF3H NOTCH1 SKIV2L BAX CD274 ZNF292 LY96 SFRP1 CDH5 ACT35 SELENBP1 MIR125A GSTT1 KMT2C EVL HGF MMP1 LAMC1 STAT1 TYMP ICAM5 RASSF1 LRRC3B PYCARD MIR29A ARID1A ACSL4 TP63 TNFSF13 CYP1B1 GATA4 VHL SLC2A1 MIR26A1 NUSAP1 DACT1 EPCAM EPHA1 EIF4G1 CD109 PRSS1 APC2 GRID1 UQCRC2 ACKR3 ERCC2 ABCA13 CASP3 EFEMP1 HAPLN1 CSF2 MIRLET7A1 PMS2 MIR335 PARP1 DHRS2 MMP11
Intersection of targets	47	PRKCE NR3C2 PLAU KDR NTRK1 NOS2 PTPRS MET PRKCB DNMT1 MDM2 AKT2 PIK3CA ALK ADORA1 IL2 CHEK1 BCL2 TOP2A IGF1R BCL2L1 PPARG TERT RELA RARB RET AURKA SRC ABCB1 PGR MTOR CYP2A6 TLR9 AKT1 PDGFRA CA9 PTGS2 ESR1 CDK4 P2RX7 PIK3CD FLT4 ESR2 AKT3 IL6 RPS6KB1 MMP9

Table S4. The major targets of xanthones for colon cancer treatment and the topological parameters.

Uniprot ID	Gene name	Target name	Degree	Betweenness	Closeness
P12931	SRC	Proto-oncogene tyrosine-protein kinase	25	0.294747	0.633333
P31749	AKT1	RAC-alpha serine/threonine-protein kinase	24	0.279567	0.633333
P42336	PIK3CA	Phosphatidylinositol 4,5-bisphosphate 3-kinase catalytic subunit alpha isoform	23	0.271149	0.612903
Q04206	RELA	Transcription factor p65	22	0.083746	0.542857
Q00987	MDM2	Double minute 2 protein	21	0.067354	0.513514
P42345	MTOR	Mammalian target of rapamycin	19	0.065197	0.513514
P23443	RPS6KB1	Ribosomal protein S6 kinase beta-1	18	0.052632	0.500000
O00329	PIK3CD	Phosphatidylinositol 4,5-bisphosphate 3-kinase catalytic subunit delta isoform	18	0.064466	0.506667
P03372	ESR1	Estrogen receptor	17	0.041123	0.487179
P08069	IGF1R	Insulin-like growth factor 1 receptor	15	0.040422	0.487179
P31751	AKT2	RAC-beta serine/threonine-protein kinase	13	0.040020	0.481013
O14757	CHEK1	threonine-protein kinase Chk1	13	0.038323	0.475000
P60568	IL2	Interleukin-2	13	0.014568	0.469136
P05231	IL6	Interleukin-6	13	0.011265	0.469136
P05771	PRKCB	Protein kinase C beta type	13	0.010787	0.457831
Q07817	BCL2L1	Bcl-2-like protein 1	12	0.005996	0.447059
O14746	TERT	Telomerase reverse transcriptase	12	0.005856	0.447059
Q9Y243	AKT3	RAC-gamma serine/threonine-protein kinase	12	0.005780	0.447059
P10415	BCL2	Apoptosis regulator Bcl-2	11	0.005605	0.436782
P35968	KDR	Vascular endothelial growth factor receptor 2	10	0.005630	0.436782
P08581	MET	Hepatocyte growth factor receptor	10	0.005557	0.436782

Q92731	ESR2	Estrogen receptor beta	9	0.005180	0.431818
P26358	DNMT1	DNA (cytosine-5)-methyltransferase 1	8	0.005112	0.431818
P16234	PDGFRA	Platelet-derived growth factor receptor alpha	8	0.005046	0.431818
P11802	CDK4	Cyclin-dependent kinase 4	7	0.004884	0.417582
E9PD35	FLT4	Vascular endothelial growth factor receptor 3	7	0.004693	0.408602
P35228	NOS2	Nitric oxide synthase	7	0.004580	0.395833
P06401	PGR	Progesterone receptor	7	0.004470	0.395833
P37231	PPARG	Peroxisome proliferator-activated receptor gamma	7	0.003916	0.391753
Q02156	PRKCE	Protein kinase C epsilon type	7	0.003367	0.391753
P07949	RET	Proto-oncogene tyrosine-protein kinase receptor Ret	7	0.003272	0.391753
P14780	MMP9	Matrix metalloproteinase-9	6	0.002015	0.387755
P04629	NTRK1	High affinity nerve growth factor receptor	6	0.001422	0.383838
O14965	AURKA	Aurora kinase A	6	0.001422	0.372549
Q9UM73	ALK	ALK tyrosine kinase receptor	5	0.001355	0.348624
P35354	PTGS2	Prostaglandin-endoperoxidase synthase 2	4	0.001325	0.339286
P08183	ABCB1	ATP binding cassette subfamily B member 1	3	0.001304	0.316667
P00749	PLAU	Urokinase-type plasminogen activator	3	0.001304	0.294574
P10826	RARB	Retinoic acid receptor beta	3	0.001294	0.285714
P11388	TOP2A	DNA topoisomerase 2-alpha	3	0.000869	0.277429
Q9NR96	TLR9	Toll-like receptor 9	3	0.000769	0.265383
P08235	NR3C2	Mineralocorticoid receptor	3	0.000669	0.263571

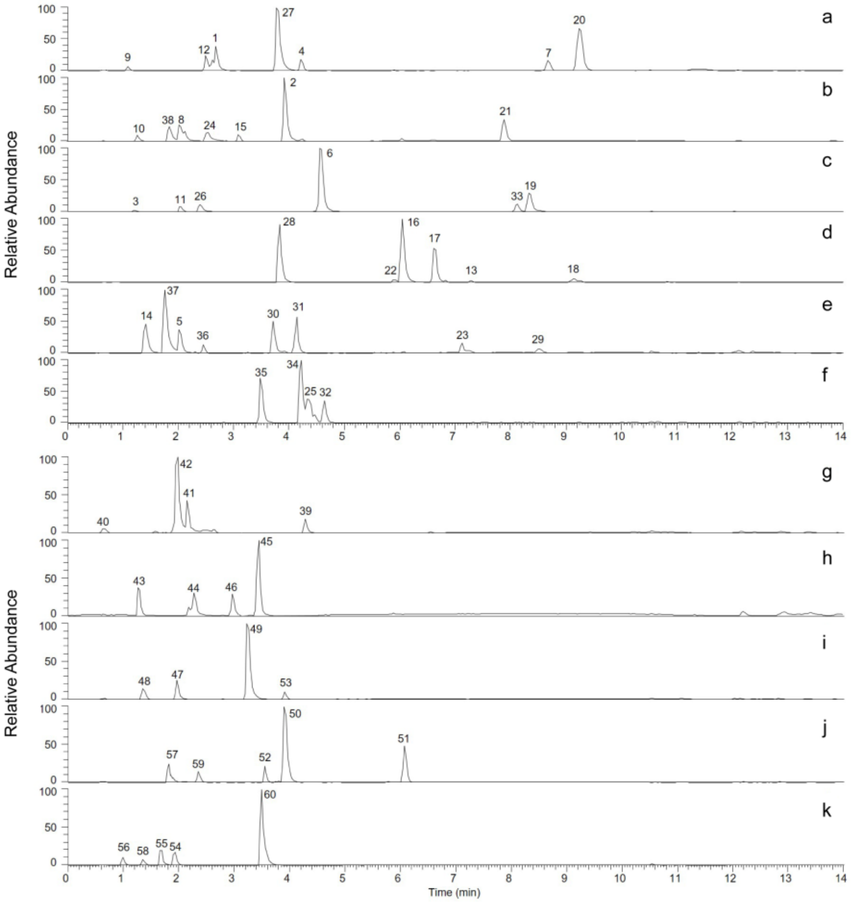

Figure S1. BPC for the mixed references.

a: The mixed reference of compounds **1**, **4**, **7**, **9**, **12**, **20** and **27**; b: The mixed reference of compounds **2**, **8**, **10**, **15**, **21**, **24** and **38**; c: The mixed reference of compounds **3**, **6**, **11**, **19**, **26** and **33**; d: The mixed reference of compounds **13**, **16–18**, **22**, **28**; e: The mixed reference of compounds **5**, **14**, **23**, **29–31**, **36** and **37**; f: The mixed reference of compounds **25**, **32**, **34** and **35**; g: The mixed reference of compounds **39–42**; h: The mixed reference of compounds **43–46**; I: The mixed reference of compounds **47–49** and **53**; j: The mixed reference of compounds **50–52**, **57** and **59**; k: The mixed reference of compounds **54–56**, **58** and **60**.

Raw data from western blot assay

Notes:

In Figures S2-S9 of the raw data, "H" stands for HT29 cells and "L" stands for LoVo cells. Numbers 22, 23, 24, 25, 28 and 29 correspond to compounds **16**, **17**, **18**, **19**, **22** and **23**. Respectively.

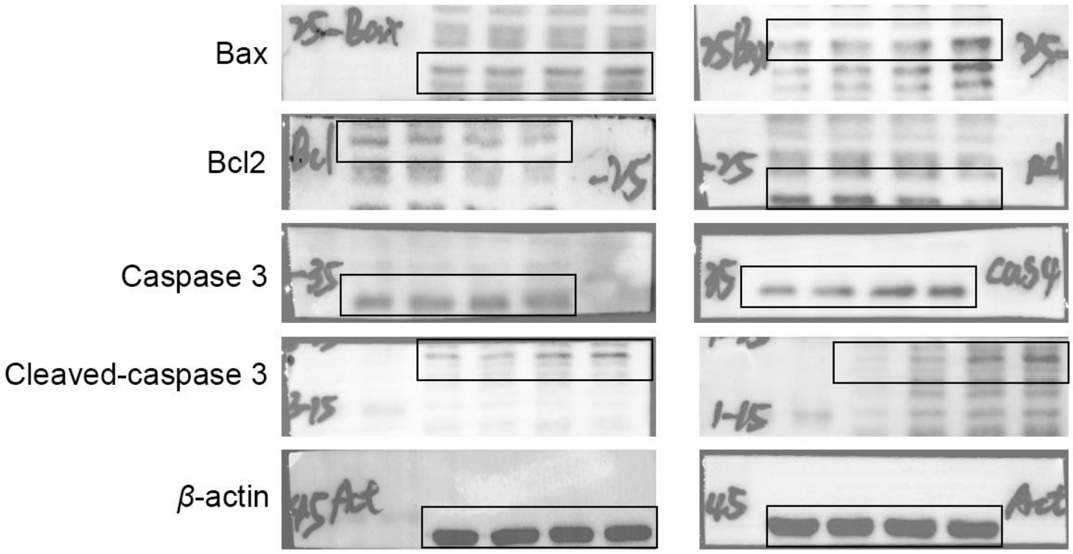

Figure S2. The raw data of Figure 3E.

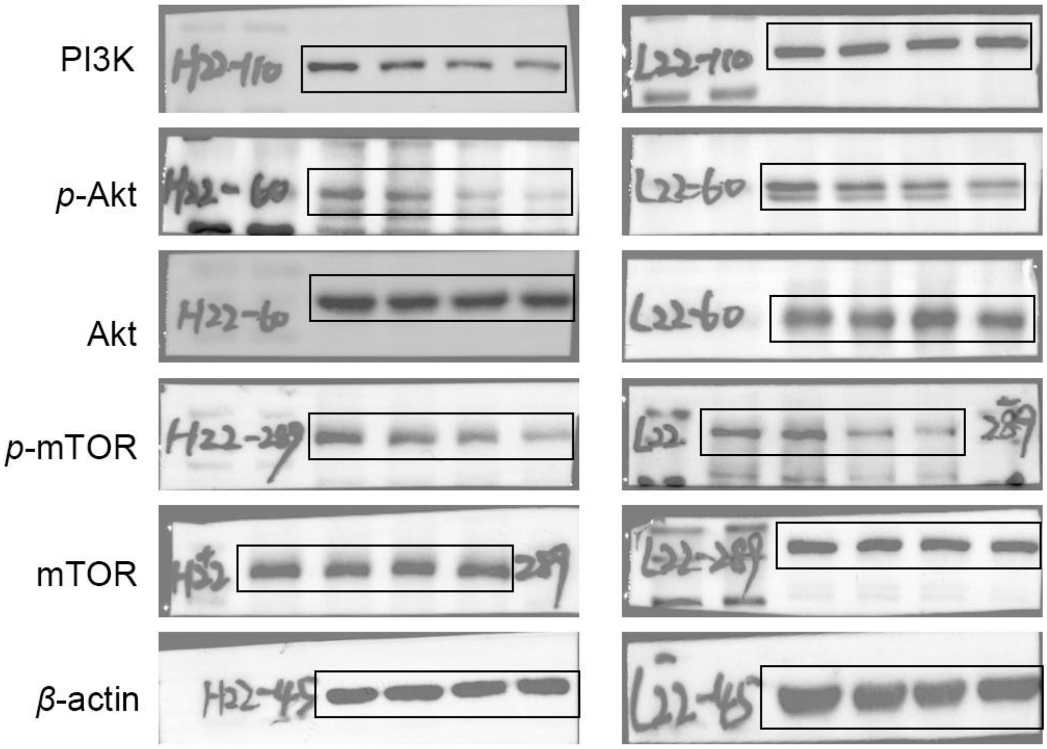

Figure S3. The raw data of Figure 7A.

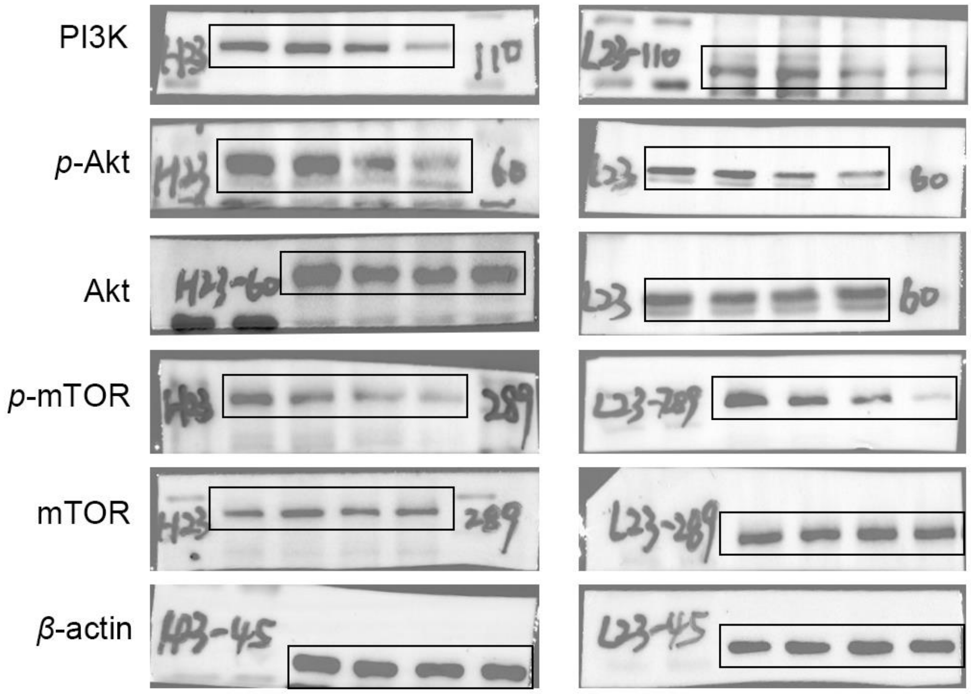

Figure S4. The raw data of Figure 7B.

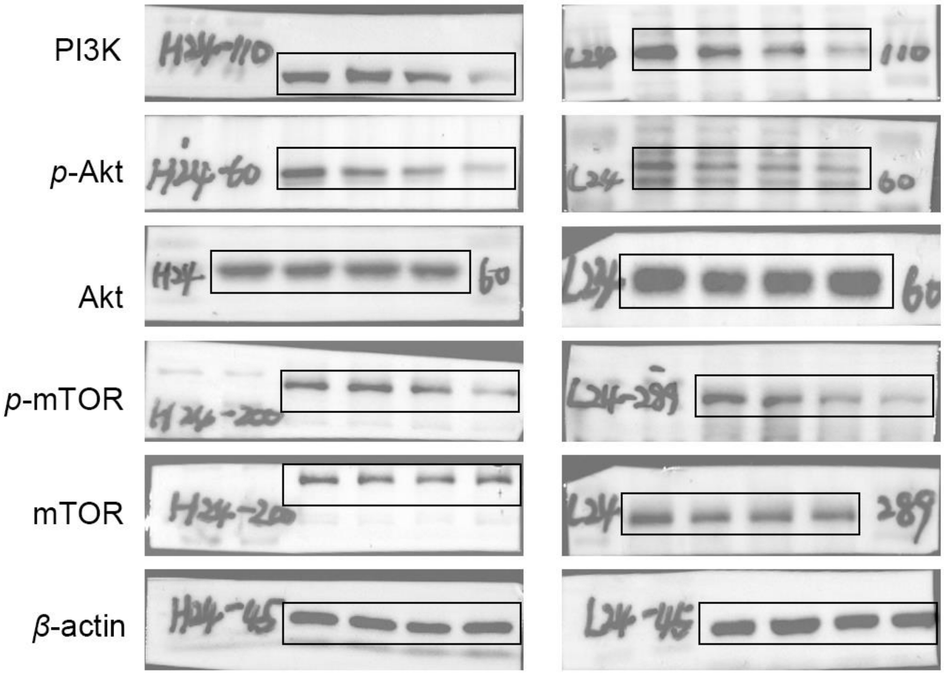

Figure S5. The raw data of Figure 7C.

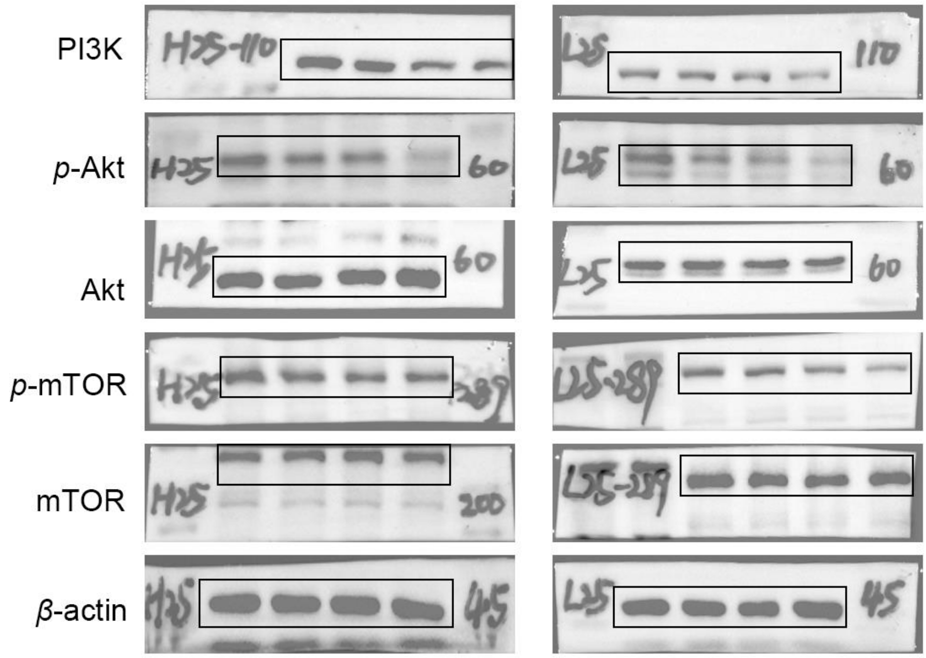

Figure S6. The raw data of Figure 7D.

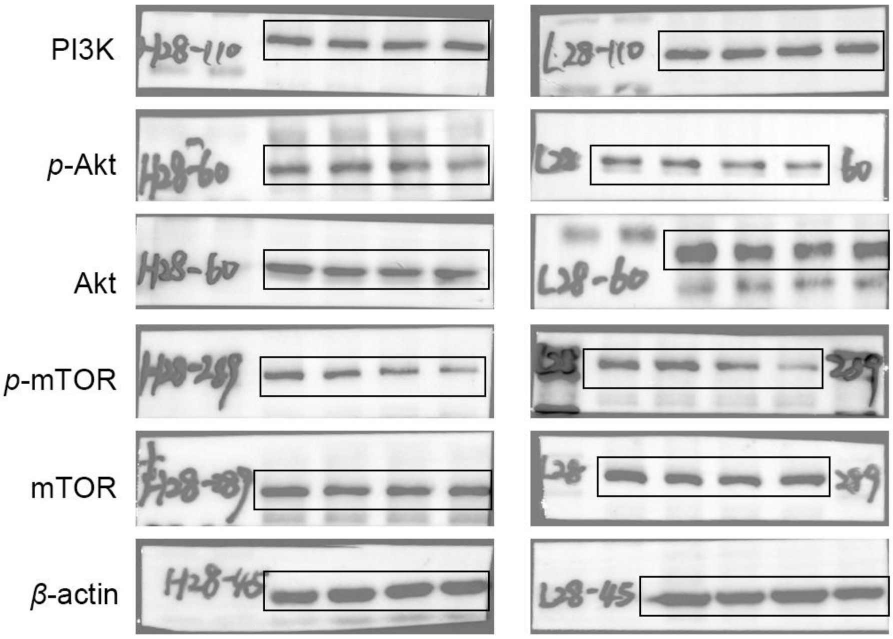

Figure S7. The raw data of Figure 7E.

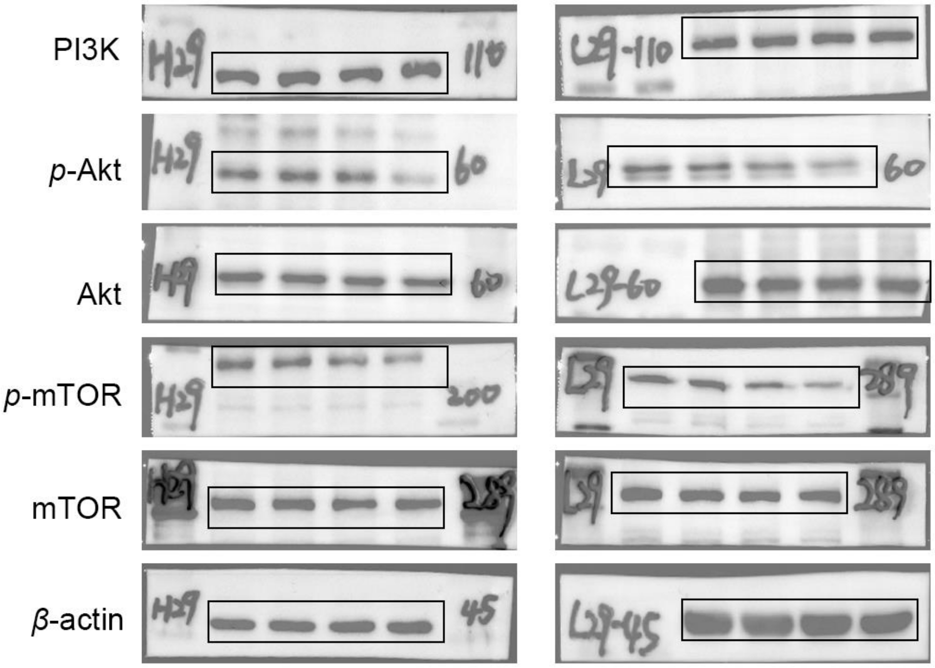

Figure S8. The raw data of Figure 7F.

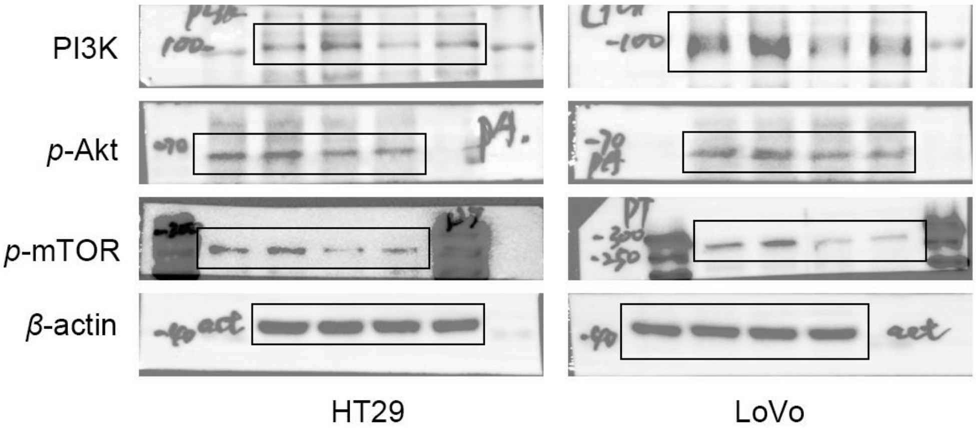

Figure S9. The raw data of Figure 8.
